# Multifunctional Structured Platforms: From Patterning of Polymer-Based Films to Their Subsequent Filling with Various Nanomaterials

**DOI:** 10.3390/polym13030445

**Published:** 2021-01-30

**Authors:** Madalina Handrea-Dragan, Ioan Botiz

**Affiliations:** 1 Interdisciplinary Research Institute in Bio-Nano-Sciences, Babes-Bolyai University, 42 Treboniu Laurian Str. 400271 Cluj-Napoca, Romania; iuliana.dragan@ubbcluj.ro; 2 Faculty of Physics, Babes-Bolyai University, 1 M. Kogalniceanu Str. 400084 Cluj-Napoca, Romania

**Keywords:** polymer film patterning, top–down and bottom–up lithography, material deposition techniques, structured platforms

## Abstract

There is an astonishing number of optoelectronic, photonic, biological, sensing, or storage media devices, just to name a few, that rely on a variety of extraordinary periodic surface relief miniaturized patterns fabricated on polymer-covered rigid or flexible substrates. Even more extraordinary is that these surface relief patterns can be further filled, in a more or less ordered fashion, with various functional nanomaterials and thus can lead to the realization of more complex structured architectures. These architectures can serve as multifunctional platforms for the design and the development of a multitude of novel, better performing nanotechnological applications. In this work, we aim to provide an extensive overview on how multifunctional structured platforms can be fabricated by outlining not only the main polymer patterning methodologies but also by emphasizing various deposition methods that can guide different structures of functional nanomaterials into periodic surface relief patterns. Our aim is to provide the readers with a toolbox of the most suitable patterning and deposition methodologies that could be easily identified and further combined when the fabrication of novel structured platforms exhibiting interesting properties is targeted.

## 1. Introduction

In the past years, a variety of conventional [1,2,3,4] and less conventional [5,6,7] micro- and nano-patterning methods have been used to generate periodic surface relief patterns. As a consequence, a huge diversity of patterned materials developed in time. These materials were shown to exhibit enhanced and/or novel properties and have been embedded in modern technological and engineering areas [8,9,10] in order to improve the quality and functionality of different devices. Nowadays, the number and diversity of such devices significantly increased inclusively due to the fact that the periodic surface relief patterns can be routinely fabricated on (polymer-covered) solid or flexible substrates and can be further filled with other (functional) materials by employing a variety of available deposition methodologies [11,12,13,14]. The resulting structured platforms (SPs), i.e., periodic surface relief patterns of certain shape and dimension that are filled in a disordered or (hierarchically) ordered manner with various materials of different size, shape, and function [15,16,17,18], are being currently used in a variety of optoelectronic [9,16,19], photonic [20], biological [17,21,22], sensing [13], or storage media [18] applications. Therefore, it is obvious that in order to further exploit such applications, we have to design and develop novel SPs with puzzling properties. This implies that we have to appropriately choose the most suitable methods and approaches related both to the patterning of polymer-covered substrates and to material deposition methodologies. To do this efficiently, we need to have at hand an overview of all the advantages and disadvantages of the above-mentioned methodologies.

**Figure 1:**
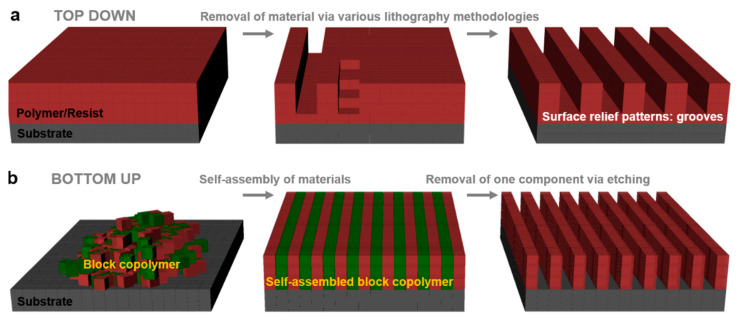
(**a**–**b**) Illustration of the top–down (**a**) and bottom–up (**b**) lithographic principles.

In general, periodic surface relief patterns of various dimensions and shapes can be fabricated using either top–down or bottom–up lithographic methodologies (Figure 1), although other methods might work as well [5,6,7]. These methodologies can be used separately [23,24,25,26] or combined [27,28]. The top–down methodology is breaking down the material system by removing molecules or atoms using electrons [29], ions [30], protons [31], light [32], etc. and leaving a clearer pattern on the surface of the material (Figure 1a), while the bottom–up methodology is based on building different blocks or stacking molecules on top of each other (Figure 1b). Both approaches have advantages and limitations and are equally important in nanotechnology and modern technological industry. The bottom–up method is supposed to be more advantageous because the resulting patterns can be of better miniature and show less defects, exhibiting a more homogenous chemical composition. Nonetheless, the top–down approach seems to display an overall better control in pattern formation on large surfaces.

In this work, we aim to provide an overview on the current polymer-based SPs and their use in nanotechnological applications, with a direct emphasis on their fabrication methodology. To accomplish this aim, in the first part of the review, we are going to offer an extensive outline about the main (polymer film) patterning methodologies, including top–down and bottom–up approaches, while in the second part of the review, we will present and analyze various deposition methods used for the guided deposition of different structures (nanoparticles, nanorods, nanospheres, etc.) of functional materials into periodic surface relief patterns. At the end of this work, readers will have at hand a toolbox of the most suitable patterning and deposition methodologies that could be easily identified and further combined in order to fabricate interesting SPs possessing puzzling properties.

## 2. Top–Down Lithographic Methodologies

Many current nanotechnological advancements rely on the development of novel micro- and nanostructured materials exhibiting good mechanical properties, flexibility, and robustness. Within this category, patterned materials displaying no chemical defects or no mechanical deformations at edges are highly desirable. Their fabrication is performed by employing different top–down patterning methods (Figure 1a) that are being continuously developed and adapted and that include photolithography, electron, proton, as well as ion beam lithography, soft lithography, scanning lithography, particle lithography, or stencil lithography.

### 2.1. Photolithography

**Figure 2:**
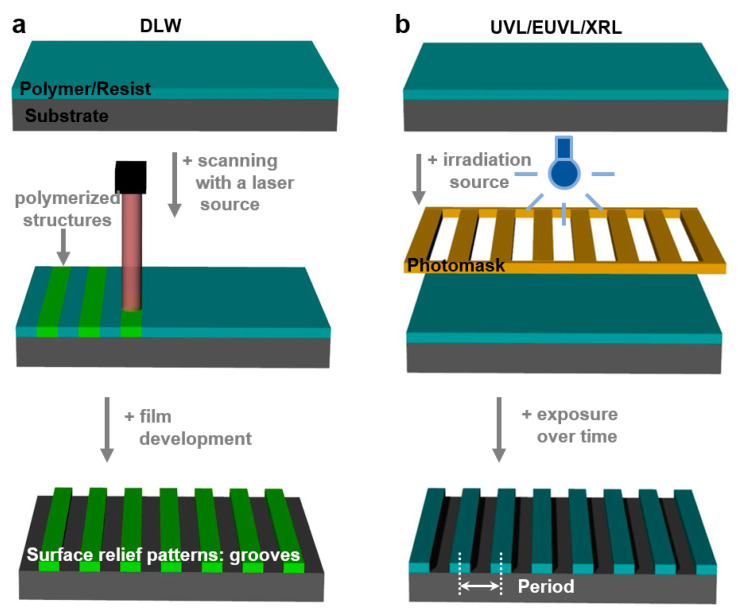
(**a**,**b**) Schematic representation of the photolithographic techniques: direct laser writing (**a**), UV, extreme UV and X-ray lithographies (**b**). Note that in (**b**), the case of patterning through the removal of material is presented.

Photolithography has proved itself to be one of the key cost-effective fabrication processes that are being used to realize surface relief structures on micro- and nanoscale in polymer-based films. This method is employing light in order to transfer a specific geometric pattern to a chemical resist that is photosensitive. Generally, while the exposure to light induces chemical changes that allow the photoresist to be removed through various development processes based on etching chemicals, the transfer of specific patterns can be realized through direct laser writing (Figure 2a) or by employing a photomask as in the case of UV lithography and X-rays lithography (Figure 2b). Here, either positive (exposed regions are etched) or negative (unexposed regions are etched) photoresists can be utilized. However, photolithography can sometimes require multiple fabrication steps, and in some situations, it can compromise the chemical and the physical properties of the materials [33]. Moreover, using photolithographic techniques on unsuitable materials is not producing high-quality surface relief patterns due to thermal changes [34] that may induce the vaporization and melting of the material or due to surface degradation and carbonization [35].

In the past years, patterned surfaces fabricated using photolithography have been used in biomedical devices [36], cell growth [37], or organic optoelectronics [38,39], just to name a few examples, and it is clear that one of the main requirements for a “healthy” development of novel applications in diverse fields of nanotechnology consists of the production of small patterned features with tailored properties and high resolution over a large area. In order to achieve such patterns through photolithography, a top-quality photomask displaying extremely fine features can be held closely to the resist material for transferring the design on the polymeric substrate by breaking down the polymer chains. Alternatively, a high-intensity laser with a well-defined wavelength can be used as well, as it can deliver optical energy to the focal spot on the material prior to its development.

#### 2.1.1. Direct Laser Writing

**Figure 3:**
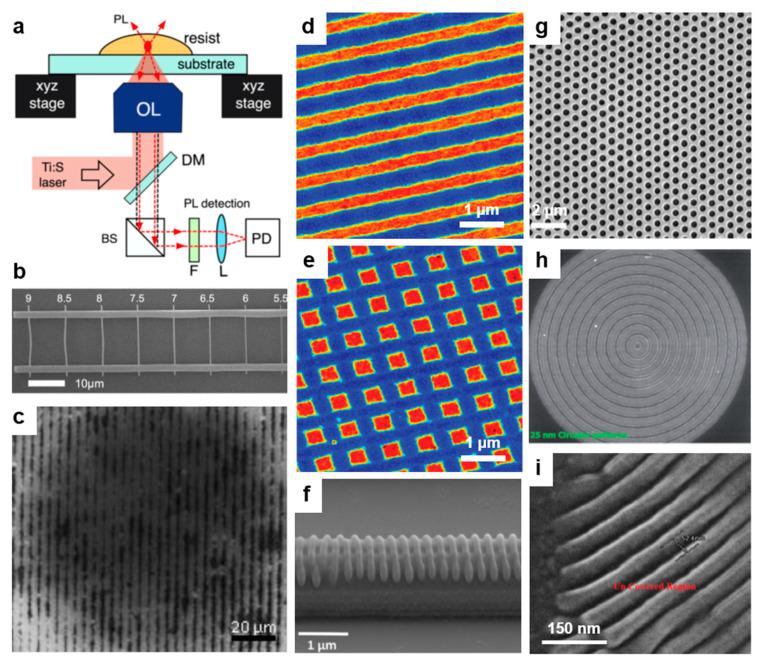
(**a**) Schematics of the direct laser writing (DLW) setup based on a photoluminescence scanning method along with its main components: an objective lens (OL), a short-pass filter (F), a dielectric mirror (DM), a focusing lens (L), and a Si photodetector (PD). (**b**) SEM image of the single lines developed by DLW. (**c**) Optical microscopy image of Ag@PMAA (poly(methacrylic acid)) obtained with 10 laser pulses. (**d**,**e**) Atomic force microscopy (AFM) images of 1D (**d**) and 2D (**e**) dedoped poly(3-hexylthiophene) (P3HT) gratings obtained by raster scanning of the patterning laser over the film. (**f**) SEM image depicting periodic surface structures sculptured by a laser on a line prepattern in photoresist. (**g**) SEM image of a nanohole pattern fabricated by UV lithography (UVL) using a 500/600 (diameter/pitch, nm) colloidal mask and an exposure time of 4 s. (**h**) SEM image of 25 nm wide circular patterns obtained via extreme UV lithography (EUVL) in non-chemically amplified hybrid (4-(methacryloyloxy)phenyl)dimethylsulfonium triflate (MAPDST)–ferrocenylmethyl methacrylate (FMMA) resist. (**i**) SEM micrograph of patterned imine-based monolayer on silicon wafers. Adapted with permission from ref. [46] (**a**,**b**), from ref. [40] (**c**), ref. [51] (**d**,**e**), ref. [52] (**f**), ref. [53] (**g**), ref. [54] (**h**) and ref. [55] (**i**). Copyright (2019, 2020) Elsevier, (2016, 2018) John Wiley and Sons and (2014) American Chemical Society. Ref. [54]—Published by The Royal Society of Chemistry.

Direct laser writing (DLW) is a photolithographic technique that can create, on a photosensitive material, permanent micro- and nano-patterns with different (hierarchical) structures exhibiting high spatial resolution (Figure 2a). Based on the multiphoton absorption process in a photoresist (which is transparent at the wavelength of the laser), DLW is properly modulating the laser until a chemical change such as polymerization of the photoresist is initiated in a small focal volume on the surface [34,40] where the laser intensity is high enough. Scanning the laser over the photoresist surface changes its solubility in the exposed regions. This procedure may be followed by the film development and leads to high-quality (periodic) surface relief patterns. The shape and dimension of such patterns can be eventually improved by employing and controlling a number of laser beams and the angle between them [41]. In addition to the laser properties, the spot size, the photoresist material, and the acceptable writing speed are also important requirements for creating a high fidelity pattern [42]. Despite the fact that this method can achieve finely patterned structures [43,44], there are cases in which the relief model is different from the design, which is inclusively due to mechanical instabilities and deformations caused by the processes of chemical development and drying or by the mechanical drifting of the focal spot position with respect to the sample [45]. However, recently, Yulianto et al. have overcome some of these limitations by replacing the conventional live monitoring of patterns based on wide-field microscopy with a photoluminescence scanning method (Figure 3a). This new setup is capable of visualizing latent three-dimensional (3D) photoexposure patterns recorded in photoresist doped with photoinitiators and exhibits a spatial resolution comparable to that of two-photon microscopy [46]. As a result, 350 nm wide single lines supported by massive walls could be demonstrated (Figure 3b).

There are other significant variable parameters to be taken in consideration when performing DLW, including the exposure time, laser power and dose, as each of them can modify the desired surface pattern [47]. For instance, the laser power has to be kept between a range of values in order to avoid undesirable local heating of the sample, which may result in damaged photoresist material. Moreover, an inappropriate laser power can alter material composition by leading to irreversible chemical bonding during the irradiation of thermoplastic polymers [48]. Furthermore, when exposed to low laser fluences, the morphology of the polymer film can induce material expansion known as swelling [49].

Another challenge related to DLW is to keep newly obtained relief patterns non-colored on the surface, since the heating or degradation of the polymer/colorant blends can induce color changing in the irradiated area, especially after the etching. Recently, Lafleur et al. overcame this limitation by presenting an improved DLW method to create desired optical patterns [50]. In this case, the common DLW technique was used on a photothermal dye, a mixture of high-density polyethylene (HDPE) and 2-(2H-benzotriazol-2-yl)-4,6-ditertpentylphenol (BZT), and it was assisted by a pulsed UV laser that controlled the local UV irradiation dose. As a result, the bulk morphology of the polymer stayed unaffected during irradiation, while the newly obtained topographical patterns appeared white on the transparent tape [50].

In recent years, DLW has been widely used not only to miniaturize the dimensions of the surface relief patterns but also to extend patterning to other materials. For example, Mulko et al. have used poly(methacrylic acid) (PMAA) doped with fluorescent silver nanoclusters in order to create groove patterns displaying an average half-peak width of 1.2 µm [40] (Figure 3c). Here, they employed 10 laser pulses of 355 nm and of 10 ns duration with a fluence of 400 mJ/cm^2^. This is impressive because in PMAA, no patterns can be created employing this wavelength. Nonetheless, using silver nanoclusters increased the absorbance at 355 nm, and PMAA patterns could be realized [40]. Another example of a system on which patterning has been successfully employed is 2,3,5,6-tetrafluoro-7,7,8,8-tetracyanoquinodimethane (F4TCNQ) doped poly(3-hexylthiophene) (P3HT). In this case, Su et al. have used 405 nm and 532 nm continuous wave lasers to achieve micrometer wide grooves [38]. Moreover, using the same doped polymer system, Jacobs et al. have further shown that patterns of sub-diffraction limit down to 200 nm could be achieved with the DLW method based on a laser scanning confocal microscope combined with doping-induced solubility control [51]. Here, well-defined 1D (Figure 3d) and 2D (Figure 3e) P3HT-based gratings with a 640 nm pitch were demonstrated.

In addition to grooves and gratings, many other patterns can be fabricated with the DLW method, including hierarchical structures. Hierarchical micropillars based on polyether ether ketone enforced with carbon fibers can be fabricated with two-beam DLW based on either 263 nm or 1053 nm laser pulses at various laser fluences [23]. Other periodic surface structures can also be developed on micrometer-sized line pre-patterns previously fabricated in photoresist films by contact lithography [52] (Figure 3f). These surface structures display a period of 195 nm and were sculptured by a laser at very low laser fluences and high pulse numbers [52]. More types of structures that can be fabricated using DLW are summarized in Table 1.

#### 2.1.2. UV and Extreme UV Lithography

UV lithography (UVL) is a patterning method widely used in engineering [1] and biotechnology [56]. It is based on the transfer of a diversity of patterns, through a positive or a negative tone, into polymer-based photoresists (most often SU-8) by employing a variety of photomasks (Figure 2b), including Cr [57] or soft [53] masks. The exposure of the photoresist to a pattern of intense UV light induces a chemical change and allows the removal of exposed or non-exposed photoresist regions with a certain solution. A significant advantage of photomasks is that they can generate sub-50 nm resolution patterns [57] when employing a UV light of 365 nm, as it was shown by Liu and co-workers. Unfortunately, most of the photomasks present a huge drawback as, very often, they can be utilized for only one patterning procedure. Fortunately, this issue can be fixed in the future either by circumventing the need for photomasks through the use of digital micromirrors [32,58] or by fabricating reusable and highly durable masks through cost-effective procedures [59]. For instance, polydimethylsiloxane (PDMS) masks are currently easy to fabricate and can create, on a large area, subwavelength patterns such as nanoholes [53] (Figure 3g).

Some very specific applications might require materials that need more energy in order to be transformed into periodic relief patterns via exposure to UV light. Therefore, UV light can be replaced with light coming from a coherent powerful source generating extreme UV wavelengths and leading to extreme UV lithography (EUVL; Figure 2b). This patterning technique is able to create periodic surface relief patterns with sub-30 nm resolution [57], including arrays of 25 nm wide grooves and circular patterns (Figure 3h) generated in a hybrid non-chemically amplified photoresist (n-CARs) based on a copolymer synthesized by reacting (4-(methacryloyloxy)phenyl)dimethylsulfonium triflate (MAPDST) and ferrocenylmethyl methacrylate (FMMA) monomers [54]. The subsequent incorporation of organometallic entities within resulting patterns was demonstrated to lead to the increased thermal profile of the hybrid material and its sensitivity toward radiation. Additional details on the patterns that can be obtained using UVL and EUVL are given in Table 1.

In both UVL and EUVL techniques, photomasks designed with different array dimensions and shape are used to transfer the desired pattern on the material by modifying the exposure time [58]. Generally, when long exposure times are needed, the patterning process becomes expensive and time consuming. In most cases, the masks cannot be reused, this being one of the disadvantages of the UVL and EUVL techniques, as already stated above. Therefore, scientists working in this field have to further focus their research on one hand toward photomask circumvention [32,58] and on the other hand to the development of reusable photomasks that do not encounter deformations and are still able to generate uniform surface relief patterns [53,59].

#### 2.1.3. X-ray Lithography

X-ray lithography (XRL) comes as a complementary method to UVL and EUVL by using short wavelengths generated by a synchrotron source. The XRL setup consists of a substrate coated with a thin light-sensitive resist layer covered with a mask that is partially or completely removed by X-ray radiation passing through, leaving behind desired patterns (Figure 2b). By employing wavelengths below 1 nm, the advantages of this method consist of the depth of focus and in the high resolution of the resulting patterns [55,60]. Unfortunately, XRL needs expensive and complex instrumentation [60], and examples of patterning via XRL are rather scarce. Most recent examples of such examples (see Table 1) include resin materials based on polystyrenebenzaldimine (PSBA) [55] or bridged poly-silsesquioxanes (BPS) [60] that can lead, for instance, to groove-like features exhibiting periodicities around 50 nm [55] (Figure 3i). More information on photolithography can be further consulted in the literature [61,62].

### 2.2. Electron Beam Lithography

**Figure 4:**
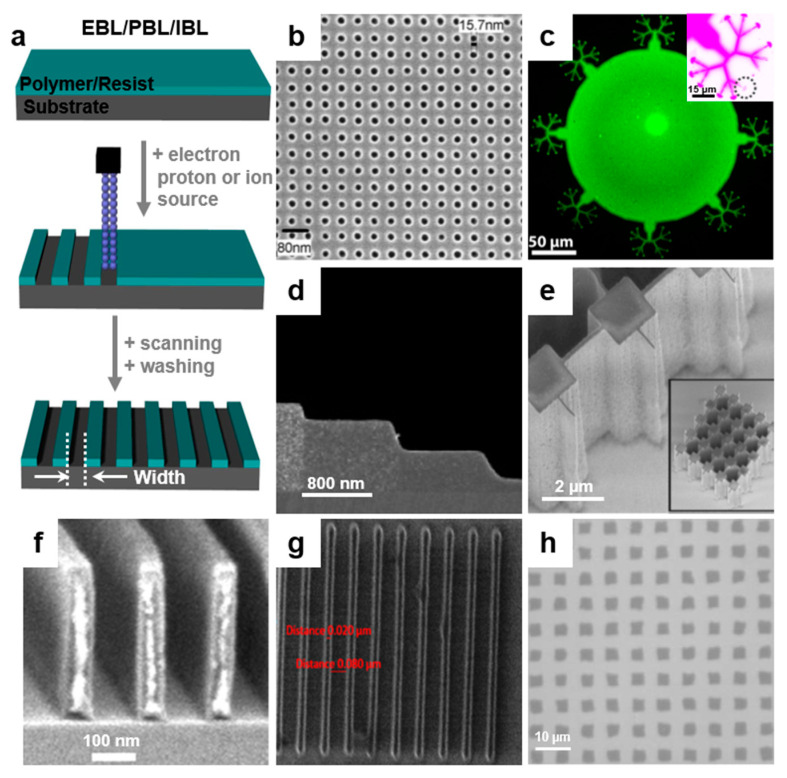
(**a**) Schematic illustration of electron, proton, and ion beam lithographies. (**b**) SEM image depicting an array of dots of a diameter below 16 nm and a pitch of 50 nm that were fabricated in a 70/90 nm thick conductive polymer/PMMA (polymethylmethacrylate) layer deposited on a fused silica substrate. Electrons of 30 keV at a dose of 125 µC/cm^2^ were employed in electron beam lithography (EBL), while a 5 nm thick anticharge layer of Cr was sputtered on top of the sample before the acquisition of the SEM image. (**c**) Confocal image of a complex spherical structure sculptured by EBL in a Teflon AF coating and filled with a fluorophore-doped lipid. Inset shows a confocal image of one of the multiple branches connected to the spherical structure. (**d**) SEM image emphasizing the cross-section through four-resist-level grayscale patterns fabricated in 2000 nm thick PMMA films. (**e**) SEM image depicting how pillars are connected to high aspect ratio walls of a width of 60 and 120 nm. Inset shows SEM image of 2 × 2 µm pillars written in a 10 µm thick SU-8 layer. The structures were written with a 1 MeV proton beam. (**f**) SEM image depicting parallel walls of a width of 50 nm written in a 350 nm thick PMMA layer by employing a focused 2 MeV proton beam. (**g**) He-ion exposed 20 nm wide lines written in a hybrid copolymer resist (100× magnification) at a dose of 120 µC/cm^2^. (**h**) Optical microscopic images showing arrays of holes obtained in PMMA films by masked ion beam lithography (IBL; irradiation was performed with 85 keV, 1 × 10^14^ ions/cm^2^ Ca^+^ ions). Adapted with permission from ref. [73] (**b**), ref. [17] (**c**), ref. [64] (**d**), ref. [31] (**e**–**f**), ref. [78] (**g**) and ref. [79] (**h**). Copyright (2011) American Vacuum Society, (2015) American Chemical Society, (2019) Robert Kirchner et al. published by De Gruyter, (2003, 2017) AIP Publishing and (2003, 2019) Elsevier.

Electron beam lithography (EBL) is a direct surface writing technique that consists of creating high-resolution patterns through the use of focused beams of electrons onto a chemical photoresist, which is often of polymeric nature (Figure 4a). In this case too, the electrons in contact with the resist are modifying its solubility, permitting the selective removal of the exposed or non-exposed regions of the resist by subsequent etching in a solvent. The main advantage of this method relies on the maskless fabrication of sub-20 nm patterns in the horizontal plane [63] and of sub-10 nm patterns in the vertical plane [64] by employing a computer software that guides a finely focused beam of electrons over the patternable surface. Nonetheless, the best contrast and sensitivity of the material has to be considered when choosing the appropriate resist for high-aspect ratio patterns [65] and when aiming at sub-20 nm resolution on both positive and negative tones resists [66]. Sometimes, depending on the resist type, EBL can also induce unwanted competing chemistries including surface-grafting or cross-linking or changes in chemical functionality [67]. For the preparation and optimization of the right resist, we advise our readers to follow suggestive examples given in the literature by Pfirrmann, Takei, and Wieberger and their collaborators [68,69,70]. They have demonstrated (see Table 1) that cardioids structures, line and space, grating, or moth-eye patterns can be fabricated by EBL. This is possible when using optimized resists based either on organic sugar-based materials derived from biomass [69] or on various copolymers exhibiting different compositions [68] and synthesized from methyl adamantyl methacrylate, hydroxyl adamantyl methacrylate, and α-gamma butyrolactone methacrylate monomers [70]. Although these resists are generally deposited via spin casting on solid substrates, there are cases when a resist is deposited through the thermal evaporation on irregular surfaces [24].

Other factors to be considered when patterning with EBL include the energy of the electrons, the material of the substrate, or the post exposure temperature. Their inappropriate use may lead to unwanted irradiation defects, resist heating effects or charging effects. Such effects, which can be predicted inclusively through simulations [71], need to be eventually eliminated. For instance, when patterning fluoropolymers, the charging process during electron exposure becomes a serious issue as the insulator substrates covered with polymeric resists accumulate charges and degrade the EBL process. Nonetheless, performing EBL at variable pressure by employing an ambient reactive gas at subatmospheric pressure can mitigate charging [72]. Other anticharging schemes reported for the fabrication of arrays of dots of diameters of few tens of nm in the polymethylmethacrylate (PMMA) resist (Figure 4b) include the use of overlayers of either aluminum or water-soluble conducting polymers, as well as sandwiching of Al or Cr thin films between the substrate and the PMMA [73].

To emphasize the power of EBL in the fabrication of various well-defined and high-quality patterns, we present below several relevant examples. Although the sub-micrometer lateral patterning of conjugated polymers such as poly(9,9-di(2-ethylhexyl)-fluorenyl-2,7-diyl) (PF 2/6) was demonstrated a decade ago [74], EBL has been recently employed to pattern wires of conducting polymers with the aim to explore their electric conductivity and quantum effects [75]. The fabrication was performed by Mahmoodian et al. by utilizing a PMMA resist combined with the conducting polypyrrole (PPy) and PPy doped with anthraquinone-2-sulfonic acid sodium salt monohydrate/5-sulfosalicylic acid dehydrate (AQSANa/SSCA). Arrays of dots exhibiting each a diameter of 100 nm and arrays of long PPy wires exhibiting 130 nm in width were obtained [75]. Shorter organic molecules forming crystals such as quaterthiophenes are also good candidates for performing EBL. Grating patterns with a period of 80 nm and 20 nm wide lines can be designed and produced on such organic crystals when using a specific exposure dose [76].

Furthermore, EBL can be used to build 3D micro- or nanostructures that can become tools for modifying the cellular shape or for controlling cell migration [2]. Vinje et al. have carefully established control over the spatial resolution and fabrication process within films of SU-8 polymer-based resist and have obtained arrays of high-aspect ratio lines and pillars with a horizontal periodicity of ~100 nm [2]. Other more complex polymeric patterns for biological purposes can be sculptured by EBL [17]. For example, spherical patterns of few hundreds of micrometers in diameter containing many branches of a lateral size down to 50 nm were demonstrated in Teflon AF coatings (Figure 4c) and employed in experiments related to phospholipid monolayer spreading and behavior in ultraconfined spaces [17].

Finally, it is worth mentioning that EBL can also be employed to create vertical grayscale patterns down to sub-10 nm step height [64]. Recent examples include fabrication in PMMA of 6 nm multilevel grayscale patterns [64] (Figure 4d) as well as of 3D structures with several micrometers in height and exhibiting a pie-chart shape [29]. More details on the next generation of EBL resists [66] and on the evolution of EBL technique [77] can be further consulted in the literature.

### 2.3. Proton Beam Lithography

Although EBL is being effectively used for the fabrication of sub-100 nm patterns, often electrons scatter and create unwanted features on the surface and/or edges. This limitation can be eliminated by employing proton beam lithography (PBL). This technique is similar to EBL as it employs protons instead of electrons (Figure 4a). Although the penetration depth of the protons varies with their energy, the interaction of protons with the electrons of the material is rather low and represents an advantage of PBL when creating deep line patterns or holes and pillars in polymer films (Table 1). For instance, van Kan et al. have effectively demonstrated the use of proton writing in producing high-aspect ratio pillars and pillars connected with walls (Figure 4e) in both PMMA and SU-8 resists [31]. Moreover, mostly miniaturized walls (Figure 4f) and line patterns exhibited only 50 nm and 30 nm in width, respectively. This was possible due to the fact that secondary electrons of lower energy induced by the primary proton beam have limited range and produce minimal local effects. Moreover, Cutroneo et al. showed that by employing protons of energy varying between 2 and 2.6 MeV along with an optimized fluence (50–280 nC/mm^2^), sharp edges and smooth sidewalls can be developed in PMMA films, leading to arrays of micron-sized holes [80].

### 2.4. Ion Beam Lithography

Ion beam lithography (IBL) is similar to EBL and PBL techniques, but instead of electrons, respectively protons, it employs ions to remove parts of the resist films and to create high-quality periodic relief patterns (Figure 4a). IBL is used in different polymeric substrates where the proximity effect limitation that would be induced by EBL needs to be alleviated. IBL is capable of creating well-defined sub-10 nm features when employing high-density resists [81]. Compared to EBL, where primary electrons interact with the resist by transferring energy via elastic and inelastic collisions, IBL can reach sub-10 nm resolution due to less backscattering of ions coupled with their better penetration properties that can be adapted to higher resistance materials [82]. For example, taking into account the characteristics of the resist, Reddy et al. have fabricated sharp inner walls in a non-chemically amplified hybrid copolymer resist made through copolymerization of an organic monomer with another inorganic monomer. Using He ions, they have obtained 20 nm wide line patterns (Figure 4g) separated by different lateral distances [78].

The IBL method can further be employed to develop small structures at the surface of the poly(acrylic acid)-based bio-resists used in biomedical studies such as surface cell adhesion [83] (Table 1). In this case, the surface energy (i.e., hydrophobicity/hydrophilicity) is important, and it can be controlled by using a mask-based IBL approach. This was shown by He et al. who changed the surface property by implanting Ca^+^ and P^+^ ions in the as-spin cast PMMA film through the use of a mesh-like mask [79]. As a result, arrays of nanometer-deep and micrometer-wide PMMA holes (Figure 4h) were created and could be further used in biomedical research. Hole patterns can further be fabricated using focused IBL directly on silicon wafers by milling, with the final aim to transfer these patterns on polymeric substrates and thus to develop robust molds for nanoimprint lithography [84].

Finally, we would like to point out that each ion beam approach comes with advantages and limitations when fabricating high-quality sub-100 nm patterns. For instance, focused IBL employing slow ions of energy in the range of keV creates surface patterning resolution of sub-100 nm by modifying the surface structure, while fast ions with energy in the range of MeV produce deep penetration into a variety of substrates. Therefore, IBL can lead to a variety of patterns of desired dimensions, including sub-10 nm lines [82]. More details on IBL can be further found in the literature [30,85].

**Table 1:** Summary of various surface relief patterns that can be fabricated using photolithography and different beam-based lithographic methodologies.

Lithography	Type of Mask	Patterned Material	Resulting Pattern	Pattern Dimension	Ref.
DLW	NA	Doped P3HT	1D, 2D gratings	640 nm/pitch	[51]
DLW	NA	PMAA doped with Ag^+^	Lines/grooves	1.2 μm/half-width	[40]
DLW	NA	PI, PEEK, PEI, PC	Lines Microcavities	6.2 μm/period 7.2 μm/diameter	[41]
DLW	NA	HDPE/BZT	Lines	hundreds of micrometers/width	[50]
DLW	NA	F4TCNQ-doped P3HT	Grooves	2 μm/width	[38]
DLW	NA	PHEMA	Gratings	2.6 μm/period	[49]
DLW	NA	PEEK/carbon fibers	Hierarchical structures	1.5 μm/period	[23]
DLW	NA	PDY-132, P3HT	Lines	17 μm/width	[39]
DLW	NA	Doped SZ2080 photoresist	Lines	350 nm/width	[46]
DLW	NA	Pre-patterned photoresist	Surface structures	195 nm/period	[52]
UVL	Photomask	Keratin photoresist	Various architectures	≈3 μm/width	[1]
UVL	Photomask	Polydiacetylene	Square patches	5 μm × 5 μm	[56]
UVL	PDMS colloidal mask	5206E, ma-N photoresist	Nanoholes	500 nm/diameter	[53]
UVL	Digital mirrors	Nucleic acid	Linear/branched structures	micrometer resolution	[32]
UVL	Digital mirrors	Polymer brush	Hypersurfaces	micrometer resolution	[58]
UVL	Cr photomask	Photoresist	Lines	102 nm/period	[57]
EUVL	IMO228775 mask	n-CARs	Lines Circular patterns	25 nm/width 25 nm/width	[54]
XRL	Photomask	PSBA	Groove lines	≈52 nm/period	[55]
XRL	Si_3_N_4_ mask	BPS	Circular lines	≈266 nm/width	[60]
EBL	NA	PMMA	Grayscale patterns Horizontal patterns	6 nm/height step 32 nm/width	[64]
EBL	NA	PS evaporated resist	Lines, ratings	30 nm/half-pitch	[24]
EBL	NA	Biotinylated PEG	Pads	10 μm range	[67]
EBL	NA	Various star BCPs	Lines	66 nm/width	[70]
EBL	NA	ZEP520A resist	L-shaped lines Rectangular mesh	60 nm/pitch 80 nm/pitch	[86]
EBL	NA	SML resist	Dense gratings	50 nm/half-pitch	[65]
EBL	NA	mr-PosEBR	Grating lines Grayscale patterns	29 nm/width 240 nm/height step	[68]
EBL	NA	Teflon AF	L-shaped lines	40 nm/half-pitch	[72]
EBL	NA	Ppy/AQSANa/SSCA	Wires Dots	130 nm/width 100 nm/diameter	[75]
EBL	NA	Conjugated PF2/6	Lines	2 μm/width	[74]
EBL	NA	PMMA	Dots	16–30 nm/diameter	[73]
EBL	NA	Quaterthiphene	Grating stripes	20–500 nm/width	[76]
EBL	NA	SU-8 polymer resist	Lines Pillars	100 nm/width 250 nm/pitch	[2]
EBL	NA	Teflon AF	Grooves	50 nm/width	[17]
EBL	NA	Sugar-based polymer	Moth-eye patterns	120 nm/period	[69]
EBL	NA	PMMA	Simulated lines	2 nm/width	[71]
PBL	NA	PMMA, SU-8 polymer resists	Lines Walls Pillars	30 nm/width 50–60 nm/width 2 µm × 2 µm	[31]
PBL	NA	PMMA	Holes	1 µm × 1 µm	[80]
IBL	NA	PAA-patterned PS	Lines	100 μm/width	[83]
IBL	Ni mesh mask	PMMA	Holes	micrometer/width	[79]
IBL	NA	Hybrid n-CAR	Lines	20 nm/width	[78]

### 2.5. Soft Lithography

Soft lithography is a family of patterning techniques used to fabricate or replicate various micro- and nanoscale periodic surface relief structures (Table 2) by employing soft, elastomeric (most notably PDMS) stamps, molds, or conformable photomasks. This methodology presents various advantages, starting from the dimension of patterns that can be fabricated (around 10 nm [87]) to the relatively large area that can be patterned [88], to the possibility of performing patterning on curved surfaces [89], to the use of automatized machinery at the industrial scale [90,91], etc.

#### 2.5.1. Nanoimprint Lithography

**Figure 5:**
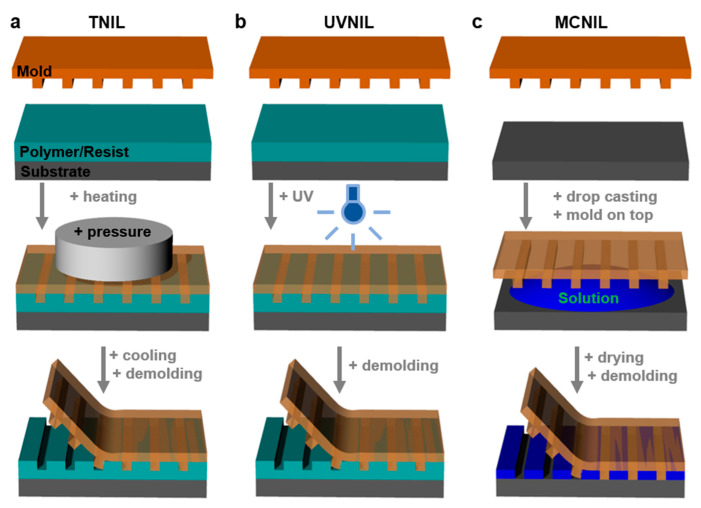
(**a**–**c**) Illustrative representation of various types of nanoimprint lithography (NIL): thermal nanoimprint lithography (TNIL) (**a**), UV nanoimprint lithography (UVNIL) (**b**), and molding in capillaries NIL (MCNIL) (**c**).

Nanoimprint lithography (NIL) is a soft lithography method consisting of a rapid, cost-effective transfer of a specific structure relief pattern from a master mold to an imprint (polymeric) resist via a variety of mechanisms, including mechano-thermal deformation (Figure 5). NIL has been widely utilized by many research groups for its high reproducibility in the realization of defect-free patterns over a large area [92] to be incorporated in sensors [93], organic solar cells [94], reverse osmosis membranes [95], and soft electrodes [96], or to develop applications in the biomedical field [97,98,99]. With NIL, different complex patterns such as elliptical hemisphere arrays [100], hole arrays [101], pillar structures [102,103], and nanogratings [104] can be achieved. Moreover, imprint molds [105] of different types have been designed, developed, and eventually improved in order to replicate high-resolution periodic patterns on various substrates with the help of various lithographic approaches derived from NIL that include thermal NIL, UV-based NIL, or molding capillaries NIL. Being a highly adaptable method, NIL can be continuously improved by considering the type of utilized resist/polymer, processing parameters, materials for molds, etc. in order to extend its productivity and applicability [106].

Thermal nanoimprint lithography (TNIL) has become a popular choice because it allows rapid patterning at low cost, and it can achieve 10 nm pattern resolution on soft and hard substrates by using PDMS molds [87]. One advantage of TNIL is that various soft and rigid molds can be re-used for several patterning procedures due to their rather acceptable lifetime (that is nonetheless limited by the constant mechanical deformation under different thermal conditions). Generally, in order to transfer a pattern from the mold to polymeric films deposited on flexible [107], solid [108], or textile [3] substrates, molds are mechanically pressed against a polymer melt [109] or films while heating the latter to melting temperatures [110,111] (Figure 5a). For efficiency reasons, it is also possible to press the molds against films soaked with non-solvents to form surface “gels” [112], or against as drop-cast solutions [113] or even to poor solutions of interest directly on the mold [114] and heat afterwards.

There are also cases when TNIL occurs at room temperature with [104] or without [115] an additional solvent annealing procedure. When no heating is needed at all, NIL becomes athermal [116]. Solvent annealing can further be combined with TNIL when the spacing between the polymer patterns needs to be increased or decreased through stretching or heating [117]. Moreover, solvent-assisted TNIL can significantly reduce feature sizes as compared to the master by controlled swelling of the patterned molds with different solvents [117]. Furthermore, TNIL can take advantage of porous gas permeable molds that increase material fluidity and thus allow patterning of materials that show poor fluidity even after being heated, such as polylactide [118].

**Figure 6:**
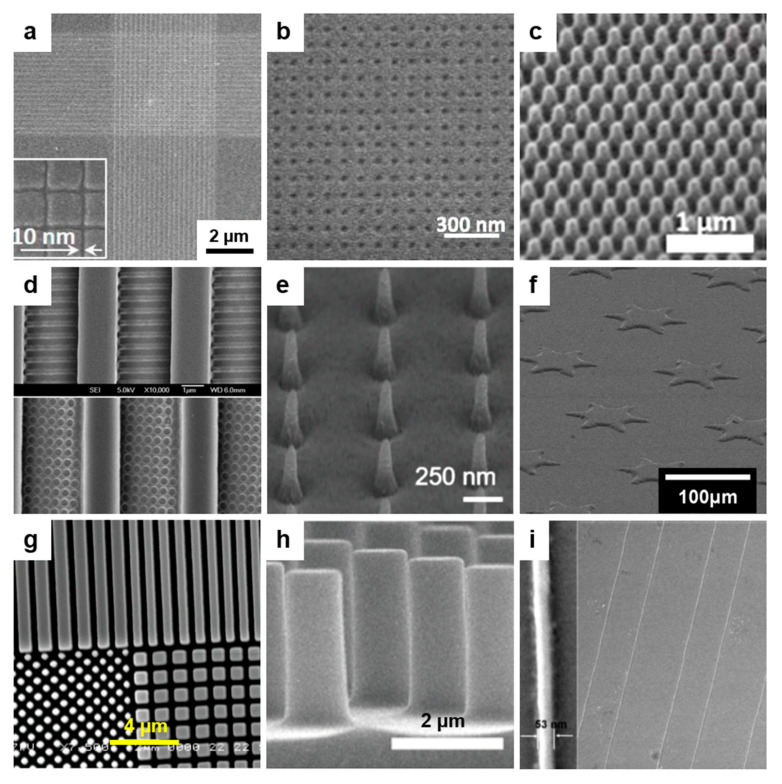
(**a**,**b**) SEM images of 10 nm wide lines (**a**) and 20 nm sized nanoholes (**b**) transferred from the mold to the poly(benzyl methacrylate) (PBMA) thermal resist. (**c**) SEM image of a moth-eye antireflective coating imprinted by TNIL on the surface of a substrate covered with a cyclo-olefin-based polymer. (**d**) SEM images of patterns obtained from a direct recessed area imprint of SU-8 polymer resist with an ethylene tetrafluroethylene (ETFE) template by TNIL showing the presence of line and space features (top) and holes (bottom) in the recessed areas of the line and space patterns. (**e**) SEM image of the 2-methacryloyloxyethyl phosphorylcholine (MPC)-grafted PEGDMA nanoneedle arrays fabricated using UVNIL. (**f**) SEM micrograph depicting patterns produced within poly(β-hydroxyl thio-ether) by UVNIL using the PDMS mold. (**g**) Various structures with critical dimension of 200 nm done by roll-to-roll (R2R) UVNIL with polymer shim (i.e., a harder formulation of the multi component UV-curable *JRcure* resist that results in a stiff but flexible layer after UV curing) at 5 m/min in a polymer stamp material *(JRstamp2* formulation). (**h**) Straight micrometer sized azopolymer pillars obtained by MCNIL. (**i**) SEM image depicting an array of 53 nm wide lines of a thermoplastic polymer fabricated on Si/SiO_2_ substrate by MCNIL using a 50 nm wide mold. Adapted with permission from ref. [87] (**a**,**b**), ref. [127] (**c**), ref. [128] (**d**), ref. [129] (**e**), ref. [130] (**f**), ref. [131] (**g**), ref. [103] (**h**) and ref. [132] (**i**). Ref. [87]—Published by The Royal Society of Chemistry. Copyright (2011) American Vacuum Society, (2016, 2019, 2020) American Chemical Society and (2010) John Wiley and Sons.

There is a variety of structures that can be obtained with TNIL (Table 2) and employed in different biological applications [111,113,114,119,120], organic photovoltaics [121], interfaces [122], superhydrophobic surfaces [107], memory elements [123], dental implants [124], etc. Miniaturized patterns obtained by TNIL and consisting in poly(benzyl methacrylate) (PBMA) lines/2D gratings and nanoholes were recently reported [87] (Figure 6a–b). The width of lines was measured to be 10 nm, while diameters of nanoholes were around 20 nm, respectively. Patterns that can be obtained using TNIL include lines of proteins [125], Nafion [126], poly(3,4-ethylenedioxythiophene):poly(styrene sulfonate) (PEDOT:PSS) [122], PMMA [123], poly(vinyl pyrrolidone) (PVP) [123], poly(vinyl acetate) (PVAc) [123], P3HT [104], or fluorinated polymer gratings [121] or azopolimeric [113] and gelatin [124] pillars, or poly(d,l-lactide-co-caprolactone) (PLCL) [111] and polyacrylamide (PAM) [119] grooves, or gelatin holes [124], etc.

Furthermore, interesting moth-eye patterns of a periodicity of few hundreds of nanometers, sculptured in cyclo-olefin-based polymers by TNIL using a PDMS stamp, were reported as good antireflective coatings [127] (Figure 6c). In particular cases, the TNIL procedure can be consecutively repeated on the same sample, but using molds comprised of patterns of smaller dimensions compared to the initial molds. At this stage, TNIL, which may or may not involve a rotation at 90° of the molds, can lead to very interesting recessed patterned structures of multiple features in a particular polymer, including the presence of line/space patterns or holes in the recessed line/space patterns [128] (Figure 6d).

UV nanoimprint lithography (UVNIL) is applying transparent molds, mostly flexible PDMS, onto various UV-curable (polymeric) liquid resists covering solid substrates. Once the mold is pressed together with the substrate, the resist is cured through its exposure to UV light and turns into a solid material (Figure 5b). This process is followed by the mold separation, which leaves behind the resulting pattern transferred into the resist [133]. When needed, the pattern from the resists can be further transferred onto the underneath substrate by employing other processes such as etching [134]. The UVNIL technique has the advantage of reproducing cost-effective yet large area patterns that are highly stable under chemical, thermal, or biological stress [135]. Nonetheless, as in the case of TNIL, when using a mold for too many patterning procedures, the patterns on the mold get deformed due to pressure and exposure to UV, leading to defects in the final transferred patterns.

UVNIL is able to produce high-quality features that can be used in biofilm [129], vascular muscle cell function [99], cell stimulation [98], data storage [136], as well as in fluidic [137] applications. Periodic patterns that can be obtained by employing UVNIL are various (Table 2) and include arrays of grooves made of inorganic–organic polymer [137], of poly(urethane acrylate) (PUA) [99], and of poly(ethylene glycol) (PEG)-based hydrogel grooves [98]. Other relief patterns are represented by arrays of 2-methacryloyloxyethyl phosphorylcholine (MPC) grafted poly(ethylene glycol) dimethacrylate (PEGDMA) nanoneedles of a periodicity of few hundreds nanometers [129] (Figure 6e), arrays of metallopolymer nanodots and nanolines [136], or even arrays of poly(β-hydroxyl thio-ether) microlines, microstars (Figure 6f), microgrids, cylindrical cavities, and other micropatterns with acute angles [130]. Again, repetitive UVNIL combined with the use of two molds comprised of patterns of different dimensions can lead to a variety of hierarchical micro–nano recessed patterned polymer structures of high aspect ratio [138].

In addition to conventional UVNIL, nowadays, the scientific community along with industrial partners has designed and up-scaled UVNIL methodologies to be able to perform patterning of various materials at the industrial scale and large area platforms [88,139]. This is known as roll-to-roll (R2R) UVNIL patterning and requires flexible yet highly resistant molds. Moreover, in order to create high-quality periodic polymeric patterns with R2R UVNIL such as arrays of lines, dots, or rectangles with critical dimension down to 200 nm [131] (Figure 6g), it is important to start with a rapid and uniform coating of resist [139]. In this sense, Koo et al. have developed a coating method based on airbrushing that was able to conformally coat the UV-curable polymeric resist at high speed and in a continuous manner, and have obtained large-area uniform lines [139]. Further information related to details on UVNIL on large industrial platforms can be found in recent reports [88,131].

Molding in capillaries NIL (MCNIL) represents another version of the NIL technique in which soft polymeric (most often PDMS) molds are placed directly on a solid substrate covered with a small quantity of liquid solution containing the desired functional material that will fill the patterns through the capillary action. Once the solvent is fully evaporated, the mold is removed, leaving behind replicated periodic patterns on the substrate (Figure 5c). MCNIL is a cost-effective, simple, and robust patterning method capable of patterning proteins as well as thermoplastic and conductive polymers at the nanometer scale on large areas [132].

The eventual slow evaporation of specific solvents makes MCNIL slower, but a slight heating of the substrate, eventually in vacuum conditions, can further compensate this drawback [102]. Instead of liquid solutions, MCNIL can also be performed on thin films. For instance, this can be done by placing the Sylgard 184 mold over PEDOT:PSS films to ensure a complete conformal contact between the two. The subsequent exposure of PEDOT:PSS films to water vapor induces swelling of the films followed by pattern replication driven by the capillary forces [140]. An interesting example where MCNIL was employed to fabricate arrays of submicrometer azopolymer pillars for unidirectional wetting and directional adhesion surfaces [103] (Figure 6h) was recently reported by Jo et al. Other examples where MCNIL is employed include the fabrication of arrays of protein lines, squares, triangles, circles, or stars for selective anti-biofouling surfaces [141]. Yet other patterns obtained by MCNIL include protein dots perfectly aligned along grooves [132] or thermoplastic (Figure 6i) and conductive polymer wires [132]. For more details, see Table 2.

#### 2.5.2. Micro and Nanocontact Printing Lithography

**Figure 7:**
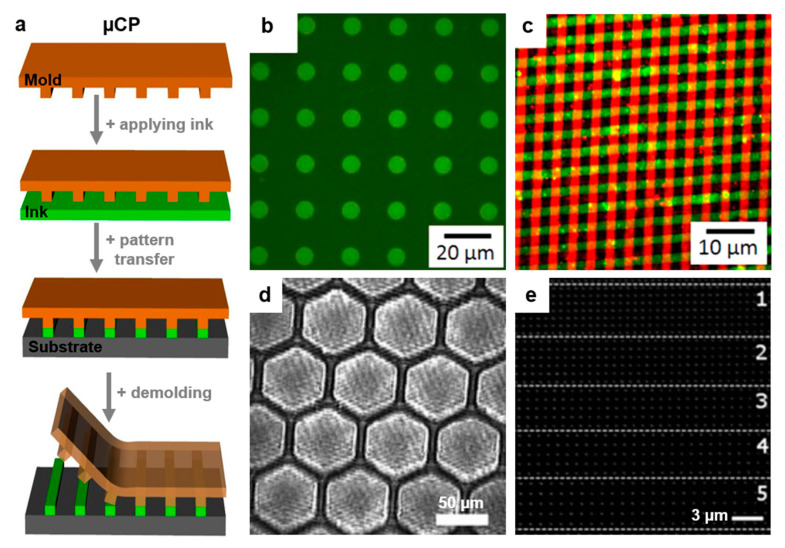
(**a**) Schematics depicting the microcontact printing (μCP) technique. (**b**,**c**) Fluorescence micrographs of micropatches of DNA (**b**) and of stripes of biomolecules (**c**) micropatterned via μCP on poly(4-aminostyrene) (PAS). (**d**) Optical image depicting polydimethylsiloxane (PDMS) micropatterns transferred to a solid silicon substrate. (**e**) Confocal fluorescence microscope image of a digital nanodot gradient patterned by nanocontact printing and comprised of grids of 200 nm dots of an “ink” made of a peptide that was mixed with an antibody. The image shows five rectangular boxes (labeled 1–5) of the digital nanodot gradient with a grid size of 1052, 1171, 1286, 1397, and 1504 nm. Adapted with permission from ref. [147] (**b**,**c**), from ref. [144] (**d**) and from ref. [152] (**e**). Copyright (2014) American Chemical Society and (2011, 2013) John Wiley and Sons.

Microcontact printing (μCP) is a soft lithographic method that creates micrometer (or nanometer, e.g., nanocontact printing) surface relief patterns onto well-defined polymeric surfaces through the conformal contact of the latter with various patterns, of a PDMS master stamp, that are covered with polymer-based or self-assembled monolayer (SAM) inks (Figure 7a). Tips and tricks on how to explore the maximum potential of the μCP technique can be found in the literature [142]. This patterning technique is preponderantly used in biological applications, and it is accompanied by processes such as functionalization [143]. In order to increase interfacial adhesion across multiple materials [144] and thus create high-quality patterns of both nano and micrometer resolution, the μCP method needs adaptation through hydrophilic surface modification of the PDMS stamps via oxidation or coating [145]. Otherwise, the hydrophobic nature of the PDMS stamp can result in the denaturation or adsorption of protein-based inks; thus, the stamp would not be sufficiently/homogeneously covered, leading to an inefficient transfer of the pattern.

The extensive use of the μCP technique in materials science, especially biotechnology [46,146] and engineering [147] applications, is well-known. Examples of efficient employment of μCP include the fabrication of patterned amyloid material with high chemical and thermal stability [46,148], other proteins [149], viral membrane clusters [150], or polymer brushes [143]. Moreover, micrometer-sized patches of DNA can be enabled by μCP on poly(4-aminostyrene) (PAS) [147] (Figure 7b). PAS can further be employed as the base on which μCP can be utilized to realize more complex structures comprised of stripes of two different types of biomolecules micropatterned laterally and vertically (Figure 7c).

The μCP method is perfectible and offers place for a continuous design and improvement of new patterns inclusively by developing a universal PDMS-assisted nanoadhesive joining technique based on the adhesive nature of a PDMS oligomer layer [144]. This technique can lead to honeycomb patterns of PDMS oligomers (Figure 7d). Moreover, nanocontact printing can be utilized in biotechnology research when printing proteins on physiologically soft substrates for cell studies [151] is desired or when the production of nanodots [152] is needed. For instance, nanodots made of peptides mixed with antibodies and exhibiting a diameter of ≈200 nm can be patterned to form digital nanodot gradients (Figure 7e) for cell haptotaxis [152]. Furthermore, this method can pattern poly(pyrrole) (PPy) nanowires with a width below 800 nm [153]. The advantages of (nano/micro)contact printing techniques include their cost-effectiveness and the possibility to adapt to nanoparticle inks and to R2R processes [154] as well as to inkless contact printing assisted by the exposure to UV light and to the use of reactive resists [155].

#### 2.5.3. Dynamic Nanoinscribing

**Figure 8:**
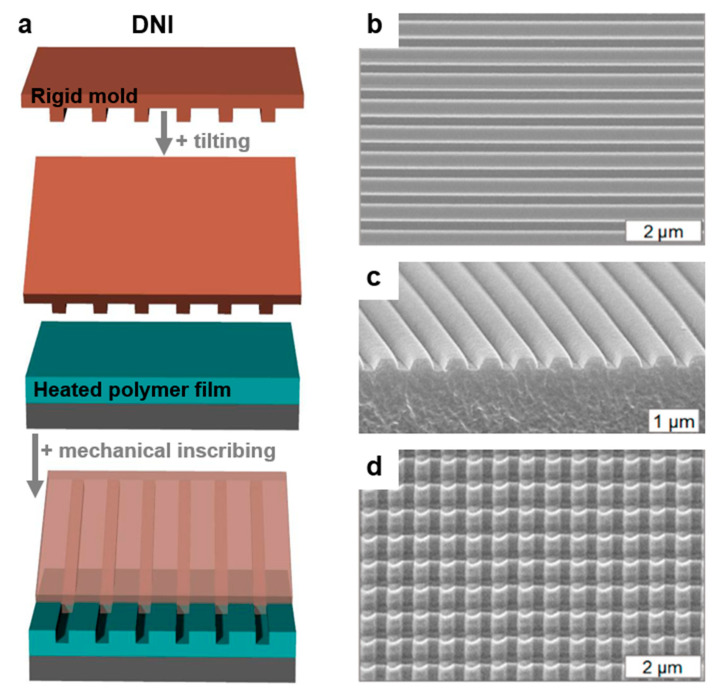
(**a**) Illustrative representation of the dynamic nanoinscribing (DNI) technique. (**b**,**c**) Top (**b**) and cross-sectional (**c**) SEM images depicting arrays of lines obtained by DNI on polymeric substrates. (**d**) SEM image depicting 2D patterns obtained by multidimensional DNI. Adapted with permission from ref. [156] (**b**–**d**). Copyright (2019) American Chemical Society.

At the end of this soft lithography section, we would like to shortly include and describe the controlled dynamic nanoinscribing (DNI) technique, even though in this case, a sliced edge from a patterned rigid mold is used. DNI is based on the continuous mechanical inscribing of various flexible polymer substrates in order to replicate different bur-free patterns (Figure 8a) with a periodicity down to ≈50 nm [156]. At this value, the experimental limit is believed to be reached. The advantage of this techniques relies on the possibility to obtain gradient patterns or multidimensional profiles by modulating the angle between the sliced edge and the flexible substrate to be patterned. The disadvantages of DNI consist of the necessity to heat the polymer substrates around their glass transition temperature (*T_g_*), while the patterns that can be obtained with DNI are rather limited to 1D arrays of lines (Figure 8b,c) or to 2D patterns obtained by multidimensional DNI [156] (in this case, DNI is performed at least two times along different directions; Figure 8d). Such patterns can be obtained on flexible polymer materials such as polycarbonate (PC), polyethylene terephthalate (PET), perfluoroalkoxy alkane (PFA), or polyimide (PI). Moreover, in order to determine the nanopattern depths and their specific profiles from rounded to angular shapes, DNI requires the precise control of several critical parameters such as the inscribing force, substrate temperature, or substrate feed rate [156].

**Table 2:** Summary of various surface relief patterns that can be developed via soft lithography.

Lithography	Type of Mold	Patterned Material	Resulting Pattern	Pattern Dimension	Ref.
TNIL	PDMS	PLCL	Linear ridges/grooves	3/3 μm/width	[111]
TNIL	PDMS	Anisotropic PAM	Linear ridges/grooves	30/30 μm/width	[97]
TNIL	Silicon	P(VDF-TrFE)	Pillars	65 nm/diameter	[110]
TNIL	PDMS	Proteins	Lines	700 nm/period	[114]
TNIL	PDMS	Poly-DR1M	Pillars	4 μm × 4 μm	[113]
TNIL	PDMS	Cyclo-olefin polymer, PC	Grooves Moth-eye	20 nm/width 347 nm/period	[127]
TNIL	PDMS	PAM hydrogel	Grooves	30 μm/width	[119]
TNIL	PDMS	Nafion resin	Grooves	800 nm/width	[120]
TNIL	PDMS	Fluorinated polymer	Gratings	760 nm/pitch	[121]
TNIL	PDMS	PEDOT:PSS	Lines	87 nm/width	[122]
TNIL	Silicon	ETFE, PMMA, SU-8	Recessed hierarchical gratings	≈254 nm/width on ~2.18 μm/width	[128]
TNIL	PDMS	PVP, PVAc, PVDF/PMMA	Lines	≥6 μm/width	[123]
TNIL	Silicon	PMMA	Gratings	250 nm/width	[108]
TNIL	PDMS	PEDOT:PSS, P3HT:PCBM	Lines	340 nm/period	[94]
TNIL	Silicon	P(VDF-TrFE-CFE)	Lines	60–100 nm/width	[93]
TNIL	Silicon	FEP, PVDF, PDFE, PFA, PCTFE	Dots	500 nm/diameter 800 nm/period	[107]
TNIL	PDMS	PS, PEDOT	Lines	416 nm/width	[96]
TNIL	PDMS	PS	Elliptical hemispheres	279 nm/width	[100]
TNIL	PDMS	Gelatin/genipin	Grooves Holes Pillars	500 nm/width 500 nm/diameter 100 nm/diameter	[124]
TNIL	Cyclodextrin-based, porous	Polylactide	Lines	150 nm/width	[118]
TNIL	PUA	HA	Lines Dots Holes	200 nm/width 480 nm/diameter 265 nm/diameter	[3]
TNIL	PDMS	PBMA	Lines, 2D grids Nanoholes	10 nm/width 20 nm/diameter	[87]
TNIL	PDMS	PLLA	Nanocups, nanocones	750 nm/pitch	[115]
UVNIL	PDMS	Metallopolymers	Nanodots	460 nm/diameter	[136]
UVNIL	Silicon	Hybrid polymer	Grooves	30 nm/width	[137]
UVNIL	Silicon	PEGDMA	Nanoneedles	50 nm/diameter (tip)	[129]
UVNIL	PUA	PUA	Ridges/grooves	800/800 nm/width	[99]
UVNIL	Silicon	PEG	Ridges/grooves	3/3 μm/width	[98]
UVNIL	PDMS	Poly(β-hydroxyl thio-ether)	Lines Stars Grids Pillars	≈70 μm/width ≈38 μm/“diameter” 10 μm/width 200 nm/diameter	[130]
UVNIL	PDMS	UV-curable polymer	Recessed pillars	300 nm/diameter	[138]
UVNIL	Bilayer h-PDMS/PDMS	Amonil/PMMA	Pillars Lines	50 nm/diameter 50 nm/width	[134]
UVNIL	PDMS	Ormostamp/Amonil	Holes	350 nm/diameter	[157]
UVNIL	Silicon	SSQ/PEG	Lines	25 nm/width	[135]
MCNIL	PDMS	mr-7010 polymer PEDOT:PSS	Nanowires	53 nm/width 100 nm/width	[132]
MCNIL	PDMS	Azopolymers	Pillars	17 μm/diameter	[102]
MCNIL	PDMS	Azopolymers	Bent pillars	1 μm/diameter	[103]
MCNIL	PDMS	PEG-PLA/PEL	Lines Squares Triangles Circles Stars	590 nm/width 10 μm × 10 μm 30 μm/size 40 μm/diameter 40 μm/“diameter”	[141]
μCP	PDMS, PVA	Protein/Sylgard 527	Nanodots	200 nm × 200 nm	[151]
μCP	PDMS	Proteins/polymer	Nanodots	≈200 nm/diameter	[152]
μCP	PDMS	Biomolecules/PAS	Stripes Pads	≈2 μm/width ≈7 μm/diameter	[147]
μCP	PDMS	Proteins/PIPAAm	Lines	20 μm/width	[149]
μCP	PDMS	Au nanorods/PLL	Grains	≈290 nm/diameter	[146]
μCP	PDMS	PPy, PI, PEN, COC	Nanowires	≈785 nm/width	[158]
μCP	PDMS	Neutravidin/PLL-*g*-PEG biotin	Spherical vesicles	≈62 nm/diameter	[150]
μCP	Rigiflex/PET	Poly(4-vinyl phenol)	Lines	400 nm/width	[155]
DNI	SiO_2_	PC, PET, PFA, PI	Lines	700 nm/period	[156]

### 2.6. Scanning Probe Lithography

**Figure 9:**
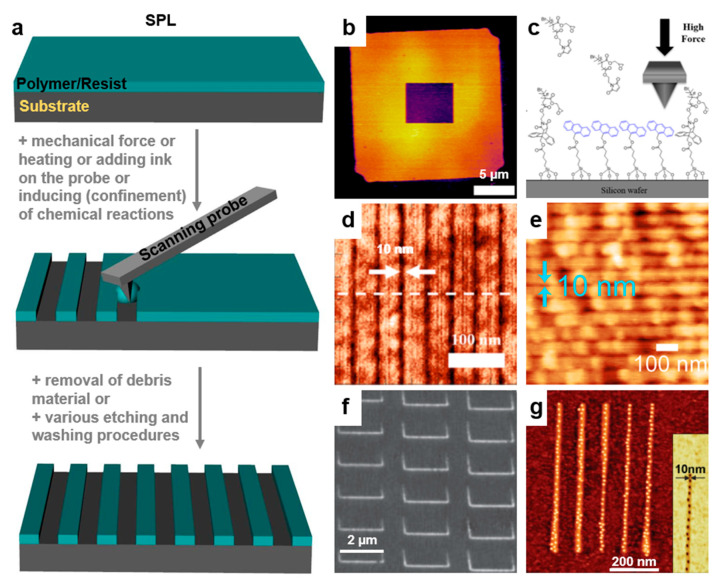
(**a**) Illustration of the scanning probe lithography (SPL) principle. (**b**) AFM topographic image of a poly-(glycidyl methacrylate) (PGMA) brush acquired under 10 nN force that was then subjected in the central region (5.46 × 4.80 μm) to high-force (450 nN) scans. (**c**) Activation of mechanophores at the interface between PGMA and silicon surface using an AFM probe. (**d**) AFM image depicting a highly uniform pattern written by thermal scanning probe lithography (TSPL) in polyacrylic acid (PAA) exhibiting 10 nm lines at <47 nm pitch. (**e**) AFM topography image showing high-resolution single-line patterns made of an enzyme on a copolymer and exhibiting a width smaller than 10 nm. (**f**) AFM image of PMMA polymer brush lines fabricated by dip-pen scanning probe lithography (DPSPL) with different operating conditions. (**g**) Typical AFM topography image of lines of ferritin molecules deposited on the 10–15 nm local oxide lines. The inset shows the AFM phase image of a single oxide line containing individual dark spot ferritin molecules. Adapted with permission from ref. [166] (**b**,**c**), ref. [170] (**d**), ref. [161] (**e**), ref. [173] (**f**) and ref. [174] (**g**). Copyright (2019) American Chemical Society, (2017) IOP Publishing and (2010, 2014) John Wiley and Sons.

Scanning probe lithography (SPL) is a methodology used to pattern various materials down to the nanoscale and beyond, to individual atoms, without the need of masks, by employing various sharp scanning probes (Figure 9a). Such probes include the atomic force microscopy (AFM) tips controlled by high-precision piezoelectric scanners. Scanning probes are able to exert, on materials that need to be patterned, various stimuli including force, heat, or electric fields, just to name a few. There are many types of SPL, and they can be consulted in the literature [159]. Generally, SPL is used over EBL or IBL because it does not involve radiation which can, in some cases, damage the surface of thin films. Moreover, SPL is a user-friendly technique, with operation of the instrumentation being made through a computer software. Nonetheless, to create topographical patterns of nanoscale (see examples in Table 3), multiple parameters such as applied voltage, air humidity, and scanning speed have to be adapted [160]. SPL can be easily employed to create patterns at ambient heat and humidity, and it can be used on nonconductive polymers by exploiting their mechanical properties. Despite these advantages, and despite being capable to reach a sub-10 nm [161,162] patterning resolution, SPL is much slower compared to other lithographic techniques. This inconvenience can be mitigated at some extent by increasing the patterning speed through the employment of multi-tip lithographic systems [163].

#### 2.6.1. Mechanical Scanning Probe Lithography

Mechanical SPL (MSPL) is based on the mechanical force exerted by a sharp tip on a (polymeric) material in order to induce the selective removal [164,165] or spatial activation [166] of the latter on the surface, with the aim to create desired surface relief patterns (Figure 9a). Obtained patterns may include PMMA ridges of a pitch varying from 30 to 100 nm [164] or large protein covered self-assembled monolayer (SAM) assays [165]. Other patterns such as squares of several micrometers developed in poly-(glycidyl methacrylate) (PGMA) brushes deposited on maleimide-anthracene mechanophores covered silicon wafers were also demonstrated [166] (Figure 9b). Here, the mechanophores were activated locally by applying high force on the AFM probes in order to deliver mechanical stimulation and to force the individual mechanophores to undergo a retro-[4 + 2] cycloaddition reaction, with the aim to form a surface-bound anthracene moiety and free PGMA [166] (Figure 9c).

Unfortunately, the limiting factor in creating sharp and yet reproducible patterns by MSPL is the stability of the tip itself, which is vulnerable to the contamination from the debris of the removed material [167] and to the deformation induced inclusively by the patterning speed. Often, to minimize such drawbacks, MSPL is coupled with additional thermal treatment of the tip [168], while the operation of contact mode AFM without active feedback increases the patterning speed in polymeric resists. As a result, high-quality patterns, including square wave patterns, or fractal carpet patterns with a pitch of 15 nm or even circle and triangle patterns with a pitch of 40 nm [168], can be fabricated.

#### 2.6.2. Thermal and Thermochemical Scanning Probe Lithography

Thermal scanning probe lithography (TSPL) uses a scanning probe with a heated tip that can efficiently remove material from a surface without the need for significant mechanical forces. The advantage of TSPL consists in overall control over the patterning depth that leads to realization, in various polymer-based resists, of high-resolution surface relief structures at a half pitch down to 15 nm [169]. Moreover, the patterning resolution with TSPL can go further down, especially when patterns such as lines are targeted. This was demonstrated by Gottlieb et al. who have patterned ≈10 nm wide lines in thin films of poly(phthalaldehyde) (PPA) [170] (Figure 9d).

Instead, thermochemical scanning probe lithography (TCSPL) uses various scanning probes to induce thermally activated chemical reactions that are modifying chemical functionalities, inclusively in (conjugated) polymers covering solid surfaces [171]. The advantage of TCSPL is that it can generate miniaturized periodic patterns (squares, rectangles, dots) of enzymes on pre-patterned copolymers containing thermally labile tetrahydropyran carbamate-protecting groups [161]. These patterns, which also include sub-10 nm lines close to enzyme molecular dimension (Figure 9e), were proved to be ideal for various nano-biodevices [161,162]. Similarly, biodevices based on proteins covered polymeric patterns can further profit from TCSPL, as it was demonstrated by Albisetti et al. who realized streptavidin squared and triangular patterns in a methacrylate copolymer presenting functional amines protected by tetrahydropyran carbamate groups [172].

#### 2.6.3. Dip-Pen Scanning Probe Lithography

Dip-pen scanning probe lithography (DPSPL) is a mask-free patterning method based on a diffusion process, i.e., on the transferring of various liquid inks from scanning probes to a range of surfaces. In this sense, DPSPL is different from the other SPL types because it adds material (ink) on top of a certain surface instead of removing material by force or by chemical reactions followed by etching. Additional capacity for thermal heating of the probe provides DPSPL with the possibility to also create patterns from solid inks. Once heated, such inks can be deposited in their liquid state, leading to advantages such as the uniformity of the resulting structures and the high reproducibility. Moreover, 2D arrays containing tens of thousands of probes can be tailored and further used to increase the patterning speed of DPSPL beyond that of EBL [27].

There are several interesting examples reported in the literature where DPSPL was successfully employed to create polymeric patterns. For instance, this method was shown to be able to rapidly create, over a rather large area, 3D brush structures such as 66 to 115 nm wide PMMA “elongated dots” and 80 to 115 nm wide PMMA nanolines [173] (Figure 9f). Furthermore, DPSPL can be used to transfer various hybrid inks based on block copolymer and metal ions in the form of ≥100 nm features on an underlying substrate. This enables a precise control over the growth and position of individual nanoparticles in situ [175]. When the polymer is removed and the metal ions are reduced through plasma etching, arrays of single crystal nanoparticles of less than 5 nm are obtained. Similarly, DPSPL can be used to pattern lipids on SAMs with the purpose to follow lipid microdomain formation [176]. Here, the obtained patterns are comprised of lipid dots displaying a diameter in the micrometer range. Further details about the recent developments on DPSPL and about the patterning strategies associated with this technique can be found in the literature [177,178].

#### 2.6.4. Oxidation Scanning Probe Lithography

Oxidation scanning probe lithography (OSPL) is a versatile patterning method based on the spatial confinement of an oxidation reaction and can be used when sub-10 nm polymeric patterns are required [179]. The good dimensional quality of patterns is owed to impeccable close-loop correction of the AFM displacement in all three dimensions. By adapting parameters such as the tip dimension and shape, the applied voltage and the environmental humidity (usually, oxidation takes place in the presence of water adsorbed to the tip), OSPL can be used to pattern a broad range of materials, including block copolymers [179] or proteins [174] deposited on oxidized polymer brush layers [179] and octadecyltrichlorosilane (OTS) or on aminopropyltriethoxysilane (APTES) SAMs [174]. As a consequence, patterns such as narrower [179] or wider [174] than 10 nm lines (Figure 9g) or even erasable circles with a line width of ≈80 nm [180] can be fabricated. Again, to increase the patterning speed, multi-tip OSPL can be developed and used [163]. More details on the OSPL patterning technique can be further consulted in the literature [181].

### 2.7. Particle Lithography

**Figure 10:**
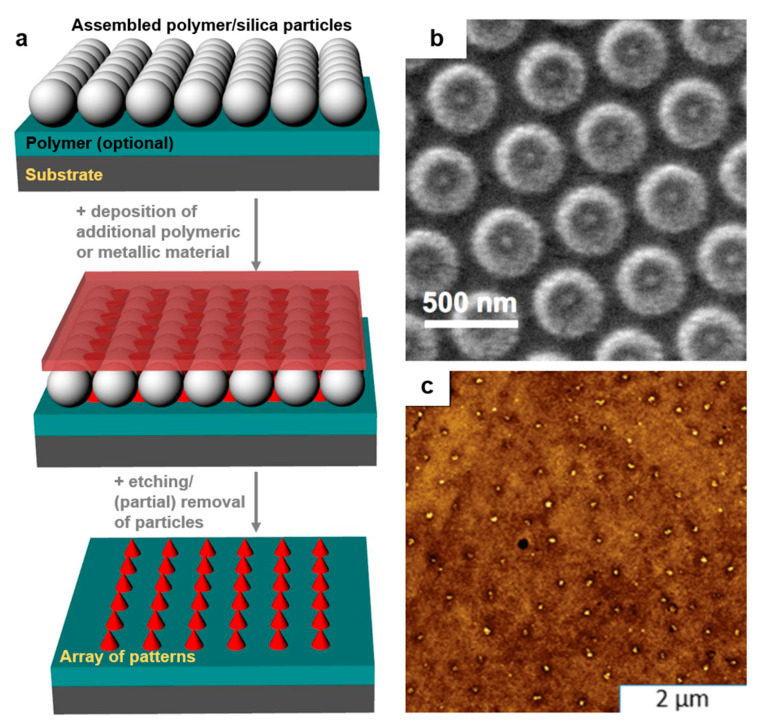
(**a**) Schematics of the particle lithography (PL) patterning technique. (**b**) Nanocraters of PAA surrounded by a matrix of poly(ethylene glycol) (PEG). (**c**) AFM topography image depicting circular patches of a protein bound to organosilane. Adapted with permission from ref. [184] (**b**) and ref. [188] (**c**). Copyright (2006) John Wiley and Sons and (2017) American Vacuum Society.

Particle lithography (PL) is a massively used patterning method that takes advantage of an evaporation mask made of (self-)assembled (colloidal) particles/spheres of silica or polymers (Figure 10a). It can constantly lead to sub-200 nm polymeric surface relief patterns of different geometrical shapes at low cost and on a large range of substrates [182]. Particles can get assembled using common techniques such as Langmuir–Blodgett, dip coating, spin coating, drop casting, doctor blade, etc. PL has the advantage of being able to develop hierarchical polymeric features in ambient conditions [183] without the need for expensive clean-room equipment. Often, this method is used along with various etching processes, including solvent [182] or (enhanced) plasma [184] etching. A typical example of patterns created by PL was given by Valsesia et al. By employing a colloidal PS mask, they have created nanocraters of PAA surrounded by poly(ethylene glycol) (PEG) (Figure 10b). Experimentally, this was done by depositing PS particles on PAA followed by the deposition of PEG on top of the PS mask, which in turn was followed by a plasma-enhanced etching process [184].

In recent examples reported in the literature (Table 3), PL has been used to create a variety of nanostructured patterns [185], including arrays of metallic nanobowls, nanoholes, nanocones, nanovolcanos, nanotriangles, etc. These patterns are obtained mostly by depositing thin metallic films on the polymeric particle mask assembled on non-confined substrates and followed by the partial removal of the mask. Nonetheless, in order to study, for example, cell recruitments or various arrangements and patterns of proteins in confined structures, microfluidic grooves can be fabricated, too. This was shown by Andersen et al. who have fabricated protein nanostructured circular domains of a diameter ranging down to 100 nm [186]. Other examples of PL being used in biomedical field applications include the realization of protein-covered circular patches of a diameter ranging from 15 to 200 nm (by using hole mask PL nanowells [187]) and the generation of circular patches of green fluorescent proteins bound to organosilane (Figure 10c). These patches have a diameter of ≈125 nm and were obtained by PL using 500 nm silica particles [188].

PL can be further applied to create masks [189] that then can be employed in other type of lithographic techniques in order to generate various patterns in polymeric materials. For instance, Friedl et al. have exploited a spin coating approach to prepare colloidal monolayers of PS particles of diameters ranging from few to several hundreds of nanometers on corresponding grating templates. Their studies revealed that smaller particles had a higher coverage capability [189], being better suitable for the mask fabrication. Moreover, Saracut et al. have created uniaxial colloidal arrays by the colloidal assembly of 500 nm PS spheres on a DVD substrate patterned with structures exhibiting a lateral periodicity of 750 nm [4]. The well-arranged, parallel, and periodic chains of particles were further covered with a thin layer of silver and employed as a lithographic mask to create metal half shells. Although in all the above cases polymer/silica particles were assembled into packed structures, there are examples in the literature where arrays of PS spheres adopting a non-close-packed structure are being used too, in order to fabricate cylindrical grooves in thick poly(4-vinylphenol) (PVP) layers [190].

### 2.8. Stencil Lithography

**Figure 11:**
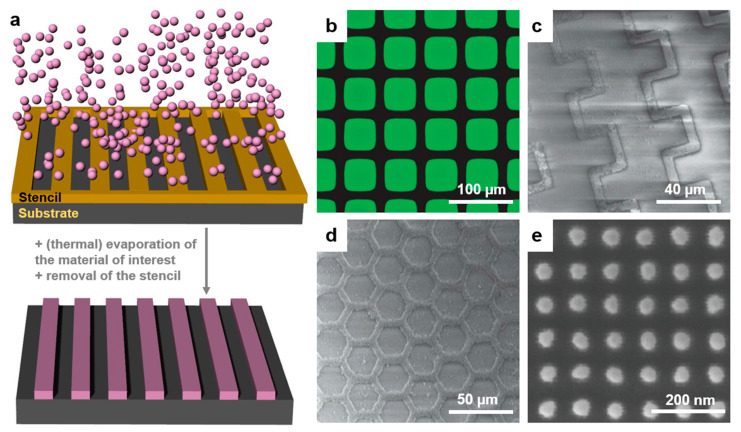
(**a**) Schematic illustration of stencil lithography (SL). (**b**) Fluorescence image of a P3HT sample after dopant evaporation and pattern development. (**c**,**d**) SEM images of zig-zag line patterns of ZnO on polyethylene terephthalate (PET) substrate with the width of line/spacing of 5.3/2.8 µm (**c**) and of a high aspect ratio honeycomb pattern on PDMS substrate with the line width of 3.2 µm (**d**). (**e**) SEM micrographs of the Au nanodots deposited via SL on polyimide. Adapted with permission from ref. [193] (**b**), ref. [194] (**c**–**d**) and ref. [191] (**e**). Copyright (2012) American Chemical Society. Ref. [193] and ref. [194]—Published by The Royal Society of Chemistry.

Stencil lithography (SL) is a patterning method based on the use of nanometer-sized apertures called shadow or stencil masks. SL is employed to create micro- or nanostructures for various applications, including plasmonics, transistors, gratings, magnetic structures, fabrication of NIL stamps, cell and protein patterning, or biosensing devices [191,192]. This method shows high versatility because it can rapidly pattern large areas, it is based on simple material deposition techniques such as thermal evaporation [193], and it can be applied on multiple materials without the need for a certain resist layer (Figure 11a). Moreover, the stencils can be reused many times, thus making SL a cost-effective patterning approach. This is especially valid when the fabrication of smaller-sized structures is targeted. Furthermore, special attention is needed when reusing the stencil, because it can become clogged, leading to lower fidelity of the initial proposed design. Additionally, the membrane stability has to be taken into consideration when the desired pattern needs a high aspect ratio (a fragile membrane of the stencil can create deformations on the pattern or it can break during the lithographic process [192]). Other advantages or disadvantages of SL were highlighted by Vasquez-Mena and collaborators and can be found in the literature [192].

An example recently reported in the literature is emphasizing the use of SL technique in the fabrication of P3HT patterns [193] for applications in energy devices (Figure 11b). Here, patterns are created by employing the selective evaporation of the F4TCNQ dopant via a shadow mask on as-spin cast P3HT films, which is followed by the development of the pattern through the dissolution and washing of undoped P3HT regions [193]. Instead of doping, other material treatment methods such as selective surface modification through oxygen plasma can be employed along with the SL technique in order to achieve micro and nanopatterning of other functional materials on flexible polymer substrates [194]. As George et al. have shown, oxygen plasma treatment is applied once a specific stencil mask is placed in contact with the PDMS substrate. As a result, zig-zag lines (Figure 11c), circular dots, nanowires, or honeycomb patterns (Figure 11d) with dimensions ranging down to 400 nm can be obtained [194]. Much smaller patterns such as high-quality lines, dots (Figure 11e), or nanowires, ranging down to 20–75 nm in size, were further demonstrated via SL on polyimide, SU-8, or PDMS, by simple vapor metal deposition through a stencil mask [191], proving that SL can be a reliable and highly robust patterning method.

**Table 3:** Examples of surface relief patterns that can be made by employing scanning probe, particle, and stencil lithographies.

Lithography	Type of Mask	Patterned Material	Resulting Pattern	Pattern Dimension	Ref.
MSPL	NA	PMMA	Ridges	30–100 nm/pitch	[164]
MSPL	NA	PPA	Fractal carpets	15 nm/pitch	[168]
MSPL	NA	PGMA	Lines	≈19 nm/period	[166]
TSPL	NA	PPA, PS-*b*-PMMA	Lines	10 nm/width	[170]
TSPL	NA	PS-benzocyclobutene)	Lines	15 nm/half-pitch	[169]
TCSPL	NA	Methacrylate-based copolymer	Rectangles Squares Lines Dots	4.5 μm × 1.5 μm 100 nm × 100 nm 8–9 nm/width 8 nm/diameter	[161]
TCSPL	NA	Methacrylate-based copolymer	Lines Squares	<10 nm/width 1 μm × 1 μm	[162]
TCSPL	NA	Methacrylate-based copolymer	Squares Triangles Lines	6 μm × 6 μm ≈11.5 μm/size <1 μm/width	[172]
TCSPL	NA	PPV	Lines	70 nm/width	[171]
DPSPL	NA	PMMA	Elongated dots Lines	≈66 nm/width ≈80 nm/width	[173]
DPSPL	NA	Lipids/SAMs	Dots	≈1 μm/diameter	[176]
DPSPL	NA	ODT/NLP 2000	Arrays of dots	3 μm/pitch	[27]
DPSPL	NA	PEO-*b*-P2VP-based	Dots	90 nm/diameter	[175]
OSPL	NA	PS-*b*-PMMA	Lines	<10 nm/width	[179]
OSPL	NA	Ferritin	Lines	10 nm/width	[174]
OSPL	NA	PMMA/PAG resist	Dots	≈100 nm/diameter	[163]
PL	PS particle mask	PAA/PEG	Nanocraters	100 nm/diameter	[184]
PL	Sulfate-latex particle mask	PDDA/PSS/PAX-XL60	Patches	200 nm/diameter	[186]
PL	Hole Mask	Streptavidin proteins	Patches	15 nm/diameter	[187]
PL	PS particle mask	PVP	Vertical cylinders	100 nm/diameter	[190]
SL	Shadow mask	SU-8, PDMS, polyimide/Au	Nanodots Nanowires	20 nm/width 65 nm/width	[191]
SL	Shadow mask	PEDOT:PSS, PC, PDMS, PET/ZnO	Dots Zig-zag lines Honeycomb array	400 nm/width 5.3 μm/width 3.2 μm/width (line)	[194]
SL	Shadow mask	P3HT/F4TCNQ	Squares	38 µm × 38 µm	[193]

## 3. Bottom–Up Lithographic Methodologies

The bottom–up lithographic approach stands on building up ordered/patterned systems starting from the molecular level by assembly processes based on chemical or physical forces that act at the nanoscale (Figure 1b). This technique implies that the hierarchical loading of block copolymer units can be controlled to create multiple desired patterns. The main lithographic methods that are bottom–up oriented are block copolymer (BCP) self-assembly and their further use as templates, DNA self-assembly, or polymer crystallization.

### 3.1. Block Copolymer Lithography Based on (Directed) Self-Assembly

Block copolymer lithography (BCPL) is a patterning method that allows BCPs to self-assemble spontaneously or via directed self-assembly into various polymeric structures of molecular dimensions [195,196,197,198] followed by the selective removal of one of the blocks, for instance via etching, in order to obtain 3D surface relief patterns [19,199,200,201,202,203,204] (note that the resulting polymeric patterns can further serve as templates for additional surface modification to obtain 3D surface relief patterns of various other non-polymeric materials; see the next section [205,206]). Here, we refer to self-assembly as to a natural nanostructure formation process based on the interplay between van der Waals forces, hydrogen bonding, and hydrophobic interactions that induces a spontaneous ordering of polymer building blocks [207,208,209]. The disadvantage of BCP self-assembly stands in the formation of defects [210]. Instead, directed self-assembly [211] uses various external stimuli (e.g., thermal annealing [197,199,209,212,213,214], solvent annealing [199,200,209,215], thermal and solvent annealing [199], ultrasounds [216], microwaves [217], magnetic fields [218], photoirradiation [219], shearing [220], addition of an ionic liquid [221], controlled spreading area [222], or epitaxy [210,223]) in order to induce and favor the self-assembly process. This process is also favored when employing click chemistry [214], and it displays a series of advantages: high-quality hierarchical [195,224] nanostructures exhibiting a low number of defects can be rapidly generated [195,213] over a large area [222].

**Figure 12:**
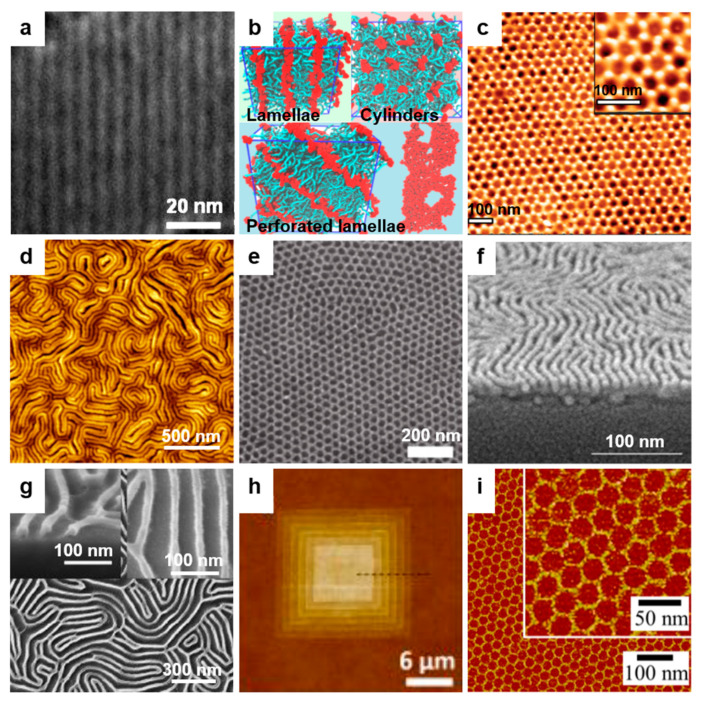
(**a**) TEM image of the bulk sample of polystyrene-*b*-poly(pentadecafluorooctyl methacrylate) (PS-*b*-PPDFMA) block copolymer (BCP) after its thermal annealing at 80 °C for 1 min. Here, bright parts of the image correspond to PS, while dark parts are representing the PPDFMA domains. (**b**) Molecular dynamics simulations depicting various mesophases (lamellae, perforated lamellae, and hexagonally packed cylinders) of high-χ block oligomers with domain sizes down to 1 nm. (**c**) AFM topographic image of a solvent-annealed PS-arm-poly(2-vinylpyridine) (P2VP)-arm-polyimide (PI) thin film treated with a CF_4_/O_2_ reactive ion etching plasma to preferentially remove the PI domains and to partially etch the P2VP domains. (**d**) AFM height images of a thin film of poly(lactide)-*b*-poly(styrene)-*b*-poly(lactide) (PLA-*b*-PS-*b*-PLA) BCP after its etching in 0.05 M NaOH alkaline solution for 30 min to remove the PLA block. (**e**) SEM image depicting patterned graphene after removal of PMMA by etching, followed by a pattern transfer of PS matrix (through a layer of SiO_2_ via a second etching procedure) and by the removal of all materials above the graphene sheet. (**f**) Tilted SEM view of 5 nm wide domains transferred on a silicon wafer via a methodology comprised of complete removal of the PVBD block by CO_2_ etching and complete removal of the chromium hard mask and carbon layer through a second Cl_2_/O_2_ etching. (**g**) TEM images depicting platinum (Pt) structures obtained after the etching of polymer matrix from the Pt-infiltrated PS-*b*-P2VP BCP thin films. (**h**) AFM image of a terraced gradient poly(ethylene oxide (PEO) polymer brush depicting a PEO brush with 5 concentric bands. (**i**) AFM image of hexagonal DNA assembled arrays. Adapted with permission from ref. [213] (**a**), ref. [228] (**b**), ref [200] (**c**), ref. [231] (**d**), ref. [234] (**e**), ref. [205] (**f**), ref. [233] (**g**), ref. [235] (**h**) and ref. [236] (**i**). Ref. [213]—Published by The Royal Society of Chemistry. Copyright (2012, 2016, 2017, 2018, 2019, 2020) American Chemical Society and (2018, 2019) John Wiley and Sons.

Although, as pointed out by Kim et al. additional challenges such as selectivity of etching or density of patterns [211] still need to be controlled and further improved; BCPL can already overcome limitations of conventional photo-, beam-, and soft lithography, and it can not only produce sub-10 nm scale features [26,214,217,219,225], but it can also lead to the fabrication of sub-5 nm relief patterns [196,199,213,226] (Figure 12a). In the future, this could be further improved as theory simulations predict the possibility to access sub-2 nm domains when employing the self-assembly of model amphiphiles [227] or even 1 nm-sized domains when making use of oligomers [228] (Figure 12b). Nonetheless, this high lateral resolution might come often with challenges such as mechanical stability [196].

Self-assembly and directed assembly are based on the microphase separation of different polymer blocks into dense structures [207]. Such separation is possible due to the minimization of the molecular interactions between different polymer species and is occurring when the enthalpic contribution is greater that the entropic contribution [229]. The resulting structures depend on the ratio of block lengths, on the strength of interaction, and on the number of blocks [230]. Since modern synthetic chemistry provides possibilities to design BCP macromolecules with specific length scales and geometries, such macromolecular systems can self-assemble in a hierarchical way on multiple length scales ranging from nanometers to macroscopic sizes, leading to a vast variety of structures [230]. Resulting BCP-based structures, mainly lamellae and vertical/horizontal cylinders (depending on the length of each polymer block in the diblock or multiblock copolymer configuration), can undergo various etching processes that will selectively remove one of the polymer blocks, leaving behind various (empty) surface relief patterns. Examples include arrays of dots [202], hole-tilings [200] (Figure 12c), and curved/straight grooves [199,202,221,231] (Figure 12d).

In the past years, BCPL was widely used in nanotechnological applications [200,208,211,217], including opto- [207] and microelectronics [228]. This method has proven itself to be a highly promising method, especially because it relies on a multitude of ordered structures obtained by directed and self-assembly such as parallel or perpendicularly oriented lamellar structures [26,219,221,225,226,228,229], (hexagonally packed) cylinders [26,199,217], semispherical [197] or body-centered cubic spherical structures [26], nanomesh structures [209], strand structures [222], double-gyroids [26], square, rectangular, and rhombic arrays of BCPs [220], quasi-hexagonal micellar structures [216], Archimedean tilings [200], and more [195,212,213,215,218,232]. Moreover, other more complex 2D [195] and hierarchical [195] or non-native [232] 3D BCP structures can be obtained via multiple self-assembly (because the fabrication of ordered structures requires an extremely precise control over ordering in BCP thin films [229], the latter can be further improved [216,218] inclusively through the use of chemical vapor deposition [201]).

Common polymeric materials that can be used to create nanostructures by self-assembly include polystyrene-*b*-poly(vinyl benzyl azide) [208], polystyrene-*b*-poly(vinyl pyridine) [233], polystyrene-arm-poly(2-vinylpyridine)-arm-polyisoprene (PS-arm-P2VP-arm-PI) [200], maltoheptaose-*b*-polystyrene [217], polystyrene-*b*-polyethylene oxide [207], and many more [195,199,209,212,215,219,221,225,226,231,232]. That is why BCPs are considered highly representative materials in innovative applications such as smart materials [211], 2D photonic crystals [200], graphene nanocomposites [202], or information storage devices [199], just to name a few. Multiple other applications of BCPs can be further consulted in the literature [26,218,225].

### 3.2. Further Use of Assembled Block Copolymers as Lithography Templates

As stated above, the directed/self-assembly of BCPs mainly leads to phase-separated, highly ordered nanodomains such as lamellae and horizontal or vertical cylinders. The removal of one polymer block through etching leads to 3D periodic surface relief patterns of the other polymer block. However, very often, such a patterning process does not stop here, and these resulting patterns are being further used as templates for subsequent pattern transfer [234,237]. This is needed in order to fabricate 3D structure relief patterns [233,238] of sub-10 nm features [205] on large areas [206,234] on various other materials/substrates.

The transfer of (flexible [237]) surface relief patterns on graphene [234,237] or other solid, often functionalized [239], substrates [206,240] can be done by employing additional etching or infiltration [233,238,241] procedures, which is followed by the controlled deposition of thin films of various materials [205,241,242], including metals [243] and metal oxides [244]. Examples of etching procedures include UV ozone [243], oxygen [241,243], nitrogen [241], or other types [239,241] of plasma etching, CO_2_ and Cl_2_/O_2_ reactive ion etching [205], etching in alkaline solutions [204]), etc. As a result, BCP templates made from materials such as poly(cyclohexylethylene)-*b*-poly(lactide) [242], polystyrene-*b*-poly(2-vinyl pyridine) [233,245], polystyrene-*b*-polymethyl methacrylate [238,246], polystyrene-*b*-polydimethylsiloxane [239,247,248], or others [205] lead to the fabrication of a large variety of versatile surface relief patterns of different material nature. Examples include (metal) nanodots [240,249,250], bimetallic NP arrays [251], perovskite cylinders, lamellae or cylindrical mesh [245], 2D metal/silicon nanowires [206,249], metal oxide arrays [242] of nanorods [244] or nanorings [243], and more [205,252].

A detailed example of a BCP system used as a lithographic template was given by Kim et al. who have employed one of the most utilized BCP system (PS-*b*-PMMA) in order to transfer arrays of vertical cylinders/holes into a graphene sheet sandwiched between an SiO_2_ layer and an SiO_2_ substrate [234]. Practically, once the vertical PS-*b*-PMMA cylinders have been assembled, the PMMA block was removed by O_2_ and CHF_3_ + O_2_ plasma etching procedures. Further etching with O_2_ plasma through the PS matrix, through the holes corresponding to former PMMA and through the underneath SiO_2_ layer and graphene, followed by the removal of all materials above graphene, led to well-patterned graphene on the SiO_2_ substrate [234] (Figure 12e). Similarly, by directing the self-assembly of poly(5-vinyl-1,3-benzodioxole-*b*-pentamethyldisilylstyrene) (PVBD-*b*-PDSS) into 5 nm lamellar domains followed by removal of the PVBD block by etching, Lane et al. have transferred the resulting surface relief patterns onto a silicon substrate by performing a second etching through an underlying chromium hard mask and carbon layer [205] (Figure 12f).

This pattern transferring methodology based on two or more etching procedures is not singular. Recently, Subramanian et al. have demonstrated a patterns transfer methodology based on an infiltration process [233]. Inorganic materials such as Pt can be infiltrated (by a liquid-phase infiltration performed at elevated temperatures) into organic templates created via the self-assembly of polystyrene-*b*-poly(2-vinylpyridine) (PS-*b*-P2VP) BCP. Once the polymer matrix is removed from the Pt-infiltrated BCP thin films by etching, various conductive Pt structures can be obtained [233] (Figure 12g). Of course, all patterns transferred with the above methods can further be employed in various applications in nanoengineering [246,249,251,252], quantum technology [247], or optoelectronics [237] in order, for example, to manufacture high-performance tribological generators [252], transistor circuitry [206], masks for lithographic purpose [206,253], photonic nanostructures [240,248], etc.

### 3.3. Polymer Crystallization as a Patterning Tool

Polymer crystallization is a process of (partial) alignment of polymer chains in bulk films or solutions under specific conditions dictated by various physical and chemical parameters, including temperature [254], pressure [255], molecular weight [256,257], chemical structure [198], solubility [258], environment [259], type of substrate [19], etc. During crystallization, polymer chains may come together folded [260] or fully extended [256] and may form ordered domains called lamellae, which in turn give birth to spherulites. Generally, polymer crystallization proceeds upon solvent evaporation, upon cooling from melting, or upon mechanical stretching, and it induces significant structural changes in the polymeric material that further affect its properties [261,262]. Therefore, crystallization is a very adaptable method that can lead to well-ordered patterns. For instance, the simplest case is to use hexamethylbenzene as a solvent to create lamellar domains in thin films of poly(l-lactic acid) (PLLA) blended with PS [263]. In this case, it is then easy to selectively remove the PS domains by the immersion of films in cyclohexane and to obtain lamellar surface relief patterns of PLLA. Other, more complex patterns can also be obtained by employing polymer crystallization. For instance, rather recently, Mei et al. have fabricated terraced (Figure 12h) and smooth gradient polymer brushes or even pyramid polymer bushes by starting with a PEO single crystal and further relying on a polymer-single-crystal-assisted-grafting-to method [235].

### 3.4. Patterning via DNA Self-Assembly

Similar to BCPs, DNA can also self-assemble into highly ordered structures, especially when a precise control is established over molecular interactions that drive atoms and/or molecules together. The advantage of using DNA self-assembly for lithographic purposes consists of the possibility of obtaining sub-10 nm biological superstructures [264], often displaying a wide range of shapes that include squares [265], rectangles [264], stars [264], triangles [264,265], and tetragonal or hexagonal arrays [236] (Figure 12i). Such structures can further be used as templates either to develop novel relief patterns or to integrate other functional materials to form structured platforms [236]. Moreover, DNA nanoarchitectures can be combined with top–down approaches in order to create patterns of a few hundred nanometers [236,264,265] that exhibit potential in biomolecular recognition and DNA interfaces [265], as well as in nanoarchitectonics [265] and patterning via DNA molds [236].

## 4. Patterning through the Combination of Bottom–Up and Top–Down Methodologies

**Figure 13:**
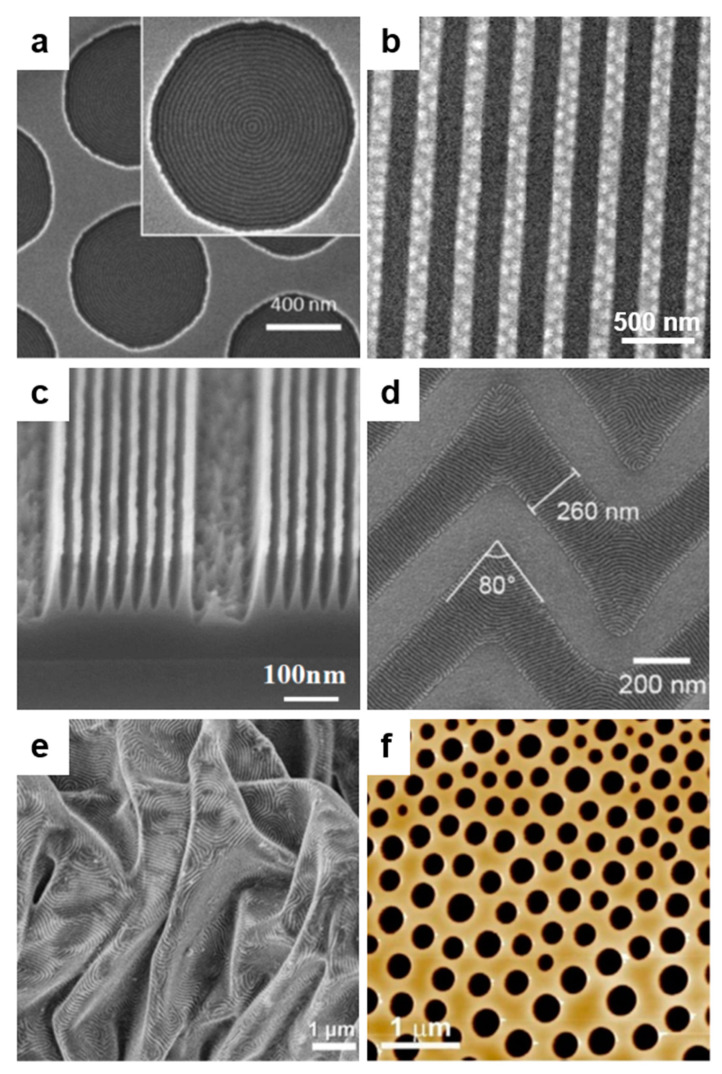
(**a**) A self-assembled concentric in-plane cylindrical pattern obtained by the directed self-assembly of PS-*b*-PDMS BCP in circular trenches of a diameter of 800 nm. (**b**) SEM image of a 23 nm thick PS-*b*-PMMA film displaying 160 nm wide dot-patterned plateau domains spaced by 160 nm wide patternless trenches assembled on a topographically modified substrate prepared by EBL. (**c**) Cross-sectional SEM image of the nanofins of a periodicity of 35 nm fabricated in a nanofin array of periodicity of 395 nm. (**d**) PS-*b*-PDMS BCP alignment into 2D wrinkles with jog angles (perpendicular BCP alignment for 10% pre-strain was maintained). (**e**) SEM image of hierarchically crumpled (two times in two orthogonal directions) lamellae of PS-*b*-PDMS BCP on chemically modified graphene. (**f**) AFM image depicting submicroscopic surface relief patterns obtained by breath figure method. Adapted with permission from ref. [219] (**a**), ref. [273] (**b**), ref. [268] (**c**), ref. [279] (**d**), ref. [280] (**e**), and ref. [281] (**f**). Ref. [268]—Published by The Royal Society of Chemistry. Copyright (2019, 2020) American Chemical Society and (2015, 2017, 2019) John Wiley and Sons.

As we have observed above, the two most common patterning methodologies, top–down and bottom–up, lead each to the fabrication of a huge diversity of periodic surface relief patterns of different material nature and functionalities. Nonetheless, in order to fabricate novel and rather unique patterns of very specific dimensions [219,266,267] over a large area [223,266], as required by many state-of-the-art applications that include optical nanoresonators [268], nanoelectronic elements [269], bioreceptors [21], transistors [270], and others [271,272,273], it is necessary to further combine top–down and bottom–up methodologies [28,205,268,269,274,275]. As a result, peculiar surface relief patterns down to the 10 nm scale [219,266,267] can be obtained through combinations of BCPL with NIL [28,205,268,269,275,276], of BCPL with EBL [270,273,277,278], of BCPL with photolithography [223,266,272], of DNA self-assembly with IBL [21], etc. Examples of the resulting surface relief patterns include 8 nm wide lines of PS-*b*-PDMS self-assembled in both straight and circular trenches [219] (Figure 13a), 10 nm PDMS spheres assembled in trenches [267], trenches with irregular features or trenches with the shape of jogs of various depths and lateral widths [273], dot-patterned domains of various heights spaced by patternless trenches of various lateral widths [273] (Figure 13b), 50 nm large polymer micelles in arrays of holes [276], arrays of DNA origami immobilized in IBL patterns [21], 100 nm wide trenches containing two lines of nanodots and nanoholes on the same substrate [272], arrays of sub-10 nm half-pitch features [269], 10 nm straight lines [277], crossed-wire nanostructures [266], etc.

An additional interesting example of patterns obtained by combining top–down and bottom–up approaches is given by the nanofins of a periodicity of 35 nm sculptured in a nanofin array of a periodicity of 395 nm (Figure 13c). These patterns were obtained by combining UVNIL (used to imprint grooves with longer periodicity in a resist) with the assembly of PS-*b*-PDMS BCP into lamellae located within the imprinted grooves that were in the meantime covered by a chromium mask. By removing PS and by etching a chromium mask and silicon, the final nanofin patterns were obtained [268]. More relevant examples on patterning with combined bottom–up and top–down methodologies can be further consulted in the literature [28,271].

## 5. Other Less Conventional Lithographic Methods

Buckling is a phenomenon that appears as a consequence of stress applied on a material. It leads to a change in material shape when the applied force reaches a critical level and induces instabilities in the material [282]. Instead, wrinkling is defined as a repeating pattern created by buckling and used for developing functional materials [283]. Examples of materials that can undergo wrinkling are poly(allylamine hydrochloride)-poly(sodium 4-styrene sulfonate) [283], silk fibroin [284], chemically modified graphene [280], PS-*b*-PDMS [279], or PDMS [7]. Multiple approaches can be followed to create periodic surface relief patterns by wrinkling, including stretch–retraction [283], reversible responsive wrinkling [284,285], controlled crumpling [280], nanowrinkling [279,285], self-organized anisotropic wrinkling [7], air plasma irradiation [7], or dynamic wrinkling [286]. The advantages of the buckling/wrinkling process are many-fold, as it is a simple, cost-effective, and yet very fast patterning method [279]. For instance, by assembling PS-*b*-PDMS BCP parallel or perpendicular to the wrinkle orientation and by manipulating the substrate strain and varying the wrinkle, periodic surface relief patterns such as 2D wrinkles with jog angles can be obtained [279] (Figure 13d). Moreover, this BCP can be further crumpled isotropically, uniaxially, or two times in orthogonal directions on chemically modified graphene in order to lead to hierarchically crumpled lamellae [280] (Figure 13e). Most of the patterns obtained using these “non-conventional” buckling/wrinkling/crumpling methods find applications in the modulation of optical transparency and thermal regulation devices [284], photonics [280], defect-tolerant devices [279], sensors [285], transparent flexible electrodes [7], biocompatible topographic matrices [282], or smart displays [286].

At the end, we would like to mention that there are other additional techniques that can be successfully used to rapidly develop materials with periodic surface relief patterns. Here, we include patterning via the breath figure method [5,281] as well as through plateau Reileight instability [6]. These techniques can lead to smooth layers with uniform nanopores [5], to partially ordered submicroscopic porous polymeric structures [281] (Figure 13f), as well as to colloidal rings [6].

## 6. Methods Utilized for Guided Deposition of Functional Materials into Periodic Surface Relief Structures

**Figure 14:**
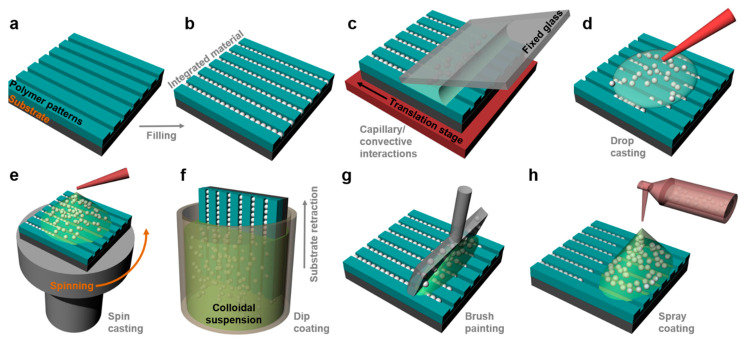
(**a**–**b**) Illustrative representation of empty (**a**) and filled (**b**) surface relief patterns. (**c**–**h**) Schematics of various deposition methods based on capillary/convective forces (**c**), drop casting (**d**), spin casting (**e**), dip coating (**f**), brush painting (**g**), and spray coating (**h**).

The introduction of various colloidal structures (including particles) into different surface relief patterns (Figure 14a–b) can lead to multifunctional SPs [4,11,287,288,289,290] exhibiting new chemical or physical properties [11,291]. Then, such SPs can be used in specific technological applications such as nanoelectronics [292], nanophotonics [292], biomedical research [22,291], and more [11,288,291,293]. To fabricate SPs, efficient deposition methods that are capable of filling the periodic nano- or micropatterns with various functional structures need to be continuously developed and improved. In this section, we review several of the most efficient and used deposition methods, including those based on convective and capillary forces, drop casting, dip coating, spin coating, spray coating, or brush painting. The size of (functional) structures/particles employed in the filling process generally ranges from several micrometers to tens of nanometers, but particles as small as 2 nm in diameter can also be used [292].

One efficient method to be employed when filling various structure relief patterns with functional materials (e.g., particles) is to employ the convective and capillary interactions [22,291,294]. Such interactions are well described in the literature [295] and can help scientists create colloidal layers by continuously [295] dragging a droplet of colloidal suspension onto a patterned substrate at a given translation speed [22,295] (Figure 14c). These interactions are highly dependent on the wetting properties [11] of the substrate. Examples of colloidal materials that can be deposited by exploiting the convective and capillary forces include gold particles [11], PS particles of various diameters [4,11,288,289,291], micro-organisms [22], and others [287,289]. Resulting (hierarchical) structures can be further used as platforms in various applications [4], including the immobilization of live organisms [22], pattern transfer [11], or building blocks for photonic elements [287].

Drop casting is another common deposition method that forms mono- or multilayered films through the evaporation of solvent, at room or at controlled temperature, from a droplet of colloidal suspension placed on a specific substrate (Figure 14d). The advantages of this method are given by its simplicity, the short experimental time, and its cost-effectiveness. Moreover, any colloidal suspension, which usually can be dropped on a patterned substrate in any desired quantity, undergoes colloidal self-assembly. Under optimized conditions, this process may lead to arrays of square-like patterns filled with self-assembled particles [296], to PS particles assembled [297] on gold-patterned arrays [12], or to PS sulfate latex particles selectively assembled inside groove and hole patterns [298]. Nonetheless, the disadvantage of this approach consists in the limited control over the solvent evaporation rate and thus over the colloidal self-assembly process. As a result, material particles can experience coffee stain effects [296,299] and can lead to inhomogeneous filling of the patterns over the substrate area. According to the literature, the filling of various surface relief patterns via drop casting can create structures highly suitable for applications in organic electronic devices [9], biosensing [12], photonics [12,298], resistive humidity sensors [300], or lithography templates [297].

Spin casting is a classical deposition technique that may be used to obtain highly uniform, often self-assembled, layers of specific material particles onto various solid substrates by rotating and spreading the corresponding colloidal suspension [290,294] with the help of centrifugal forces [301] (Figure 14e). The most important parameters considered when employing this method are the spinning speed given by the number of rotations per minute, the acceleration, and the time of spinning [189,302]. The optimization of these parameters allows the spreading of materials such as silica [14], magnetic [301], or PS particles [189,302], just to name a few, and it can transform spin casting into a versatile method that can create arrays of assembled particles [302,303] and can fill various surface relief patterns [189,301]. This latter function of spin casting can lead to the realization of structures of various geometrical shapes, including pillars surrounded by particles [14], filled holes [301], or grooves [304] that may be used in the fields of surfaces [303], engineering [301], optoelectronics [304], or plasmonics [189].

Dip coating is a deposition method that consists in placing, horizontally [19] or vertically [13,282,283,305,306], a patterned substrate into a colloidal dispersion containing particles [13,283,306,307] or other structures of interest [15], which is followed by its (motorized [306]) retraction at rather slow speeds [13,282,283] (Figure 14f). It is during the substrate retraction that particles are dragged into the relief patterns via convective/capillary forces [13]. In order for this method to be efficient, the substrate might need to remain immersed in the colloidal suspension for a shorter or longer time. Dip coating has proven itself efficient when filling with fullerene hydroxide nanoparticles specific polymeric trenches of molecular dimension [19], when self-assembling latex particles in colloidal crystals within various relief patterns [305], or when filling arrays of grooves with silica [307] and poly(*N*-isopropylacrylamide) (PNIPAM) [282] particles or even with magnetic nanorods [15], etc.

Brush painting is a deposition method that uses a (polymeric/PDMS) “brush” to spread and guide a solution or a colloidal suspension into specific surface relief patterns located on a solid substrate [308,309,310] (Figure 14g). The advantages of this method consist in the fact that it is time-effective and it can be implemented manually, over a large area, by repetitive mowing of the “brush” back and forward over the substrate. Often, if the examination of the obtained sample suggests that the degree of filling of the patterns with functional structures is not sufficient, the procedure of brush painting can resume on the same sample. This can be done by adding an extra droplet of colloidal suspension. Examples of materials that can be brush painted, besides (semi)conductive [310], dielectric [310], or active materials [308], include also nanowires [309] or colloidal particles.

There are other advanced methods to deposit structures/particles of various materials within surface relief patterns such as spray coating and Langmuir–Blodgett (LB) techniques. With the latter method, particles are initially manipulated and assembled at the air–water interface and then transferred onto a desired substrate [311]. The advantage is that LB offers control over the assembly process via parameters such as the initial particle density at the air–water interface and the substrate lifting speed during the LB particle deposition process. This method is useful in the fabrication of highly ordered and homogeneous 2D colloid crystals with non-closed-packed symmetries on micropatterned substrates [311]. Instead, the spray coating deposition approach (Figure 14h) is based on spraying solution-based materials or colloidal particles onto a desired (patterned) surface in order to fill specific patterns [8] or to change chemical or physical properties of a surface [312,313].

## 7. Multifunctional Structured Platforms (SPs) and their Applications

In the above sections, we have reviewed the recent advances of the most important top–down and bottom–up patterning methodologies that are used to fabricate substrates exhibiting periodic surface relief patterns. We have also discussed the most common deposition techniques that are generally employed to fill the resulting periodic patterns with (multi)functional structures of various materials displaying different size and shape. Furthermore, in this section, we will not only illustrate several relevant examples of (hierarchical) SPs obtained by combining patterning and filling methodologies, but we will also discuss possible applications that could integrate the resulting SPs.

**Figure 15:**
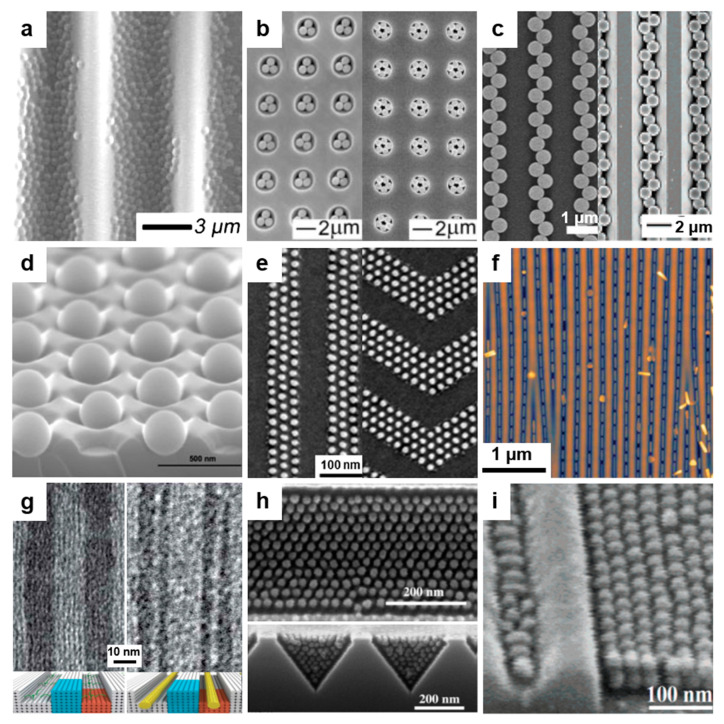
(**a**) SEM image showing PNIPAM particles assembled in buckling PDMS grooves. (**b**) SEM images depicting an array of trigonal planar clusters formed within 2 µm large cylindrical resist holes by integrating 0.9 µm large PS particles (left) and an array of pentagonal rings formed within 2 µm large cylindrical resist holes by integrating 0.7 µm large PS particles (right). (**c**) SEM images showing colloidal zig-zag arrays of PS particles on O_2_-plasma pretreated striped PS films (left) and helical chains of PS particles within V-shaped grooves (right). (**d**) SEM image displaying a tilted view of the ordered template occupied 50% with PS particles deposited by convective assembly. (**e**) SEM micrographs showing particles of PS-*b*-PDMS directly self-assembled in 1D straight (left) or jog-like (right) grooves patterned in the resist. (**f**) AFM image of an array of gold nanorods self-assembled by dip coating in the wrinkle PDMS template. (**g**) TEM images and schematics (insets) depicting 3D DNA nanotrenches of a 24 nm pitch without (left) and with (right) assembled carbon nanotube (CNT) arrays. (**h**) Top view and cross-section view SEM images of an array of PS-*b*-PFS particles showing a close-packed particle arrangement on the top surface (top) and the non-epitaxial top layer above the face-centered cubic arrangement of the lower layers (bottom). (**i**) SEM image depicting a tilted view of a 1D array of spherical and ellipsoidal particles of PS-*b*-PFS within a groove exhibiting a width near the one-row to two-row transition. This image emphasizes the comparison between the out-of-plane dimensions of confined and unconfined domains. Adapted with permission from ref. [282] (**a**), ref. [287] (**b**), ref. [13] (**c**, left) and ref. [288] (**c**, right), ref. [315] (**d**), ref. [18] (**e**), ref. [15] (**f**), ref. [317](**g**), ref. [318] (**h**), and ref. [319] (**i**). Ref. [15]—Published by The Royal Society of Chemistry. Copyright (2003, 2006, 2016) American Chemical Society, (2011) Cambridge University Press, (2020) The American Association for the Advancement of Science, (2013) Shuaigang Xiao et al. published by SPIE and (2003, 2009) John Wiley and Sons.

The most common material used for the filling of various surface relief patterns is represented by structures of spherical shape such as PS [13,282,287], silica [287,307], gold [282,314], or BCP particles [18], just to name a few. However, PNIPAM particles can also be used to fabricate arrays of particles assembled in buckling polymer grooves (Figure 15a) when applications in optoelectronics and sensing are targeted [282]. A variety of more or less complex structures can be designed and developed using spherical particles. For instance, Xia et al. have assembled PS and silica particles in various configurations, including trigonal and pentagonal (Figure 15b) or hexagonal rings, zig-zag chains, tetrahedrons, square pyramidal clusters, within arrays of prism-shaped patterns, or rectangular grooves, or pyramidal cavities, or cylindrical holes of different geometries [287]. Moreover, more complex assemblies such as (double-layered) structures both in cylindrical holes as well as in U- and V-shaped grooves along with 2D lattices of PS particles were demonstrated [287]. In addition to double-layered zig-zag chains [287] and zig-zag arrays of PS particles [13] (Figure 15c left), there is also possible to develop helical chains of PS particles with different chiralities [288] (Figure 15c right).

PS and silica particles can also be deposited via convective assembly in a non-closely-packed fashion right onto substrates covered with cubic arrays of hole surface relief patterns [315]. Moreover, at low particle concentrations, arrays of ordered PS particles occupying every other site in the template can be obtained (Figure 15d). Furthermore, the approach used to fabricate the above structures can be employed to produce 2D and 3D ordered colloids of a range of structures. Such colloids could be used in the fabrication of various photonic elements. Similar non-closely packed assemblies were also demonstrated for arrays of SiO_2_ particles deposited atop of hole polymer patterns [298].

Specific applications might demand further coating/doping of particles/patterns with other nanoparticles. This is the case when ordered silica spheres coated with silver nanoparticles are required or when arrays of voids in polymer doped with silver nanoparticles need to be developed. These structures can be used as surface-enhanced Raman scattering (SERS) substrates, in nanofabrication and sensors, or in the control of the crystallization process [316]. Control over other processes such as the self-organization of PS particles on silicon substrates can be established when employing, along with convective forces, patterning with periodic pillars of a specific diameter displayed at an optimum pitch distance [297]. As a result, the long-range ordering of self-organized PS particles between pillar patterns can lead to single crystal structures that are 100 times larger than the ones developed on non-patterned silicon substrates.

Other SPs are comprised either of arrays of 100 nm sized semiconducting methyl substituted ladder-type poly(para-phenylene) (Me-LPPP) spheres assembled into parallel grooves [9] or of globally and locally packed nanodot arrays made of 2D pillar/hole-tone or 1D straight or jog-like grooves filled with self-assembled spheres of PS-*b*-PDMS BCP [18] (Figure 15e). While the first type of structures can be used as light-emitting platforms in polymer-emitting devices [9], the nanodot arrays can be used for bit-patterned disks in the data and servo zones, respectively [18]. Moreover, such structures can be utilized as templates to transfer the patterns into quartz, with the aim of creating nanoimprint molds and further realizing high-density magnetic storage media [18].

Furthermore, arrays of parallel line patterns, obtained on graphene substrate via the self-assembly of polystyrene-*b*-poly(4-vinylpyridine) system hydrogen bonded to a small molecule called 3-pentadecylphenol, can be filled with gold nanoparticles. The resulting SPs can be employed to tackle the structure–property correlations underlying nanoparticle/graphene and small molecule/graphene nanocomposites, with the goal to develop applications such as solar cells, sensors, or catalysts [202]. These applications can also be targeted when introducing gold nanoparticles in PS-*b*-PEO templates [207].

In addition to spherical particles [9,318] or metallic nanoparticles [207,320], other anisotropic particles such as rods [15], triangles [320], or fullerenes [19] can be employed to fill various structure relief patterns and to create novel SPs. For instance, Mayer et al. have filled arrays of parallel grooves (obtained by wrinkling process) with gold nanorods (Figure 15f) by using the dip-coating technique. They have demonstrated that such structured assemblies could be transferred and further used to fabricate functional optical metasurfaces on macroscopic areas [15]. The resulting metasurfaces are capable of exhibiting an optical response in the effective magnetic permeability. Since the approach used for patterning is lithography-free, arrays of grooves filled with gold nanorods could also find application in subwavelength waveguiding and photovoltaics [16]. In comparison to gold nanorods, gold nanoparticles can be directed within DNA origami triangles (which in turn were deposited inside rectangular patterns created by EBL) to fabricate sub-10 nm components over macroscopic areas that could be used as biological platforms for smart release materials and for nanoscale electronics and photonics [320]. We believe that this range of applications might be further broadened due to the possibility of also placing other shapes of individual DNA within various surface relief patterns [321]. For example, recently, it was shown that specific DNA bricks can be assembled to form DNA brick crystal-based periodic nanotrenches displaying a uniform pitch down to ≈10 nm. By employing noncovalent interactions, the DNA antihandles can be further wrapped onto carbon nanotubes (CNTs). Then, resulting DNA-wrapped CNTs can be aligned within the tiny DNA nanotrenches (Figure 15g), leading to an SP that can find applications in ultrascaled technology nodes [317].

Sometimes, active macromolecules [8], lipids [17], or structures such as live micro-organisms [22] can also be assembled within specific surface relief patterns in order to fabricate eccentric platforms that can target novel applications. For instance, structured arrays of living yeasts and fungal spores trapped within square-like patterns of PDMS show huge potential in shaping future bio-experiments on living cells [22]. Meanwhile, solutions of lipids [17] and of active materials [8] can be used to fill the EBL- and NIL-sculptured surface relief patterns in order to fabricate platforms that can be used for the phospholipid monolayer spreading experiments [17] or for the fabrication of efficient photovoltaic devices [8].

Generally, U-shaped grooves are the most utilized patterns for filling with various functional structures. Nonetheless, V-shaped grooves can also find interesting applications. As a matter of fact, V-shaped grooves were shown to favor the self-assembly of spheres of PS-*b*-polyferrocenyldimethylsilane (PFS) BCP into well-ordered face-centered cubic packing [318] (Figure 15h). The resulting SPs may provide a useful geometry for BCPL. Nonetheless, the realization of such platforms might strongly rely on the relationships between the ordered packing of spheres within grooves and their size and aspect ratio, as well as on the dimensions of the grooves. This was further indicated by Cheng et al. when developing well-controlled 1D arrays of spherical and ellipsoidal particles of PS-*b*-PFS (Figure 15i) to be used as masks for the fabrication of various devices [319]. Similar arrays, containing one, two, three, or four bilayers of PS latex particles within a single polymer wrinkled groove further revealed the relationship between the packing of spheres and the dimension of spheres and grooves [283]. Additional information on SPs can be further consulted in the literature [20,289,322].

## 8. Conclusions

In the first part of this work, we have reviewed recent advances of the most important patterning methodologies, including top–down and bottom–up lithographic techniques that are used to fabricate periodic surface relief patterns. Relevant examples from the literature have shown that highly miniaturized relief patterns of top quality can be obtained over a large area in polymeric films by selecting the most suitable experimental parameters corresponding to each lithographic technique. While surface relief patterns of a lateral resolution down to few hundreds of nanometers can be obtained by almost any lithographic technique, fabricating patterns exhibiting a lateral periodicity of about 10 nm can be reached by EBL, IBL, NIL, SPL, or BCPL. Furthermore, the realization of relief patterns of a lateral periodicity down to 5 nm is possible by employing BCPL, with the emphasis that this resolution could go in the future down to even 1 nm, as it is currently predicted by the theory simulations performed on block oligomers.

In the second part of this review, we have emphasized the most common deposition techniques that are capable of guiding nanomaterials of various size, shape, and functionality into periodic surface relief patterns. We conclude that each deposition method is of particular interest, as in each case, the filling efficiency varies with the nature of both the surface relief patterns and of the nanomaterials used for filling the patterns. At the end, we have also illustrated the broad field of applications based on (hierarchical) SPs fabricated by guiding functional nanomaterials within various surface relief patterns. Our work can be used by readers not only to rapidly identify the most suitable patterning and deposition methodologies in order to fabricate SPs, but also to explore new “patterning–filling” combinations that could lead to the novel design and development of the future SPs.
